# Identification of a Nine-Gene Signature and Establishment of a Prognostic Nomogram Predicting Overall Survival of Pancreatic Cancer

**DOI:** 10.3389/fonc.2019.00996

**Published:** 2019-09-27

**Authors:** Mengwei Wu, Xiaobin Li, Taiping Zhang, Ziwen Liu, Yupei Zhao

**Affiliations:** Department of General Surgery, Peking Union Medical College, Peking Union Medical College Hospital, Chinese Academy of Medical Sciences, Beijing, China

**Keywords:** gene expression omnibus, nomogram, overall survival, pancreatic cancer, The Cancer Genome Atlas

## Abstract

**Background:** Pancreatic cancer is highly lethal and aggressive with increasing trend of mortality in both genders. An effective prediction model is needed to assess prognosis of patients for optimization of treatment.

**Materials and Methods:** Seven datasets of mRNA expression and clinical data were obtained from gene expression omnibus (GEO) database. Level 3 mRNA expression and clinicopathological data were obtained from The Cancer Genome Atlas pancreatic ductal adenocarcinoma (TCGA-PAAD) dataset. Differentially expressed genes (DEGs) between pancreatic tumor and normal tissue were identified by integrated analysis of multiple GEO datasets. Univariate and Lasso Cox regression analyses were applied to identify overall survival-related DEGs and establish a prognostic gene signature whose performance was evaluated by Kaplan-Meier curve, receiver operating characteristic (ROC), Harrell's concordance index (C-index) and calibration curve. GSE62452 and GSE57495 were used for external validation. Gene set enrichment analysis (GSEA) and tumor immunity analysis were applied to elucidate the molecular mechanisms and immune relevance. Multivariate Cox regression analysis was used to identify independent prognostic factors in pancreatic cancer. Finally, a prognostic nomogram was established based on the TCGA PAAD dataset.

**Results:** A nine-gene signature comprising MET, KLK10, COL17A1, CEP55, ANKRD22, ITGB6, ARNTL2, MCOLN3, and SLC25A45 was established to predict overall survival of pancreatic cancer. The ROC curve and C-index indicated good performance of the nine-gene signature at predicting overall survival in the TCGA dataset and external validation datasets relative to classic AJCC staging. The nine-gene signature could classify patients into high- and low-risk groups with distinct overall survival and differentiate tumor from normal tissue. Univariate Cox regression revealed that the nine-gene signature was an independent prognostic factor in pancreatic cancer. The nomogram incorporating the gene signature and clinical prognostic factors was superior to AJCC staging in predicting overall survival. The high-risk group was enriched with multiple oncological signatures and aggressiveness-related pathways and associated with significantly lower levels of CD4^+^ T cell infiltration.

**Conclusion:** Our study identified a nine-gene signature and established a prognostic nomogram that reliably predict overall survival in pancreatic cancer. The findings may be beneficial to therapeutic customization and medical decision-making.

## Introduction

Pancreatic cancer is lethal and aggressive with a 5-year survival rate of only 2–9% (1). Despite its low incidence, pancreatic cancer is the fourth leading cause of cancer-related death in the United States. Its mortality is increasing for both genders and it is expected to become the second most common cause of cancer-related death by 2030 after lung cancer and surpassing colorectal and breast cancers (2). Surgical resection is the only curative treatment and it significantly improves the five-year survival rate to 20–30%. However, only <20% of all patients are eligible for resection as most patients are diagnosed at an advanced stage when there is metastasis (3). Poor prognosis is caused by the rapid progression, early metastasis, and lack of typical clinical presentation or sensitive screening methods for early-stage pancreatic cancer (4). Neoadjuvant therapy, radiotherapy, chemotherapy, targeted molecular therapy, and immunotherapy have been used for treatment and have achieved certain therapeutic effects. However, for individual patients, the survival benefits of these treatments are questionable and side effects occur. Pancreatic cancer should be managed by individualized systemic treatment, which may prolong survival and improves quality of life. Therefore, an effective prediction model is needed for the accurate assessment of patient's prognosis. In this way, efficacious treatments may be selected to balance side effects and survival benefits and to decide whether to administer more aggressive treatment. Clinicopathological parameters such as AJCC TNM staging have been used for predicting prognosis of patients (5). The advancement of tumor molecular biology has facilitated the development of new prediction tools based on prognosis-related genes. These prognostic markers reflecting tumor progression at molecular level may be beneficial to realize individualized survival predictions with better accuracy.

Advances in gene chips and high-throughput sequencing have demonstrated that prognostic gene signatures at the mRNA level are able to predict overall survival in pancreatic cancer. Birnbaum et al. proposed a 25-gene signature based on clinicopathological and gene expression data that predicts post-operative overall survival independent of classical factors and molecular subtypes (6). Raman et al. reported a five-gene prognostic model (*ADM, ASPM, DCBLD2, E2F7*, and *KRT6A*) that accurately predicts overall survival from the TCGA PAAD dataset (7). Yan et al. identified a survival-related four-gene signature (*LYRM1, KNTC1, IGF2BP2*, and *CDC6*) significantly associated with progression and prognosis of pancreatic cancer (8). In-depth exploration of the public datasets (GEO and TCGA etc.) may reveal other prognostic-related genes and establish a reliable prognostic gene signature which, in combination with clinicopathological parameters, may be a powerful tool for predicting prognosis of pancreatic cancer and individualized treatment.

Here, we integrated seven pancreatic cancer datasets from the GEO database to identify differentially expressed genes (DEGs). Univariate and Lasso-Cox regression analyses were applied to identify overall survival-related DEGs and propose a prognostic gene signature based on gene expression and clinical data from the TCGA PAAD dataset. The prognostic gene signature was validated with external datasets. The molecular mechanism and tumor immunity relevance of the gene signature and its potential in guidance of immune therapy were also investigated. Independent prognostic factors of overall survival were identified by multivariate Cox survival analysis. A prognostic nomogram incorporating the prognostic gene signature and clinical prognostic factors was established to predict overall survival. Overall, our prognostic gene signature and nomogram may accurately predict overall survival of pancreatic cancer.

## Materials and Methods

### Acquisition of Gene Expression and Clinical Data

The mRNA expression and clinical data for pancreatic ductal adenocarcinoma were searched and downloaded from the Gene Expression Omnibus (GEO) (https://www.ncbi.nlm.nih.gov/geo/) using the keywords “pancreatic cancer,” “PAAD,” and “pancreatic adenocarcinoma.” “Homo sapiens” and “Expression profiling by array” were included in the next round of screening. “Cell line” and “xenograft” were excluded from the search. The gene expression microarray datasets GSE71729, GSE62165, GSE62452, GSE28735, GSE15471, GSE16515, and GSE32676 were selected and downloaded for DEG analysis (9–15). The datasets met the following criteria: (1) human pancreatic tissue samples; (2) tumor- and non-tumor pancreatic control tissue samples; (3) ≥30 samples. GSE57495 had 63 tumor tissues that were downloaded with their associated follow-up information for subsequent validation of the prognostic gene signature (16). Probes were matched to the gene symbols using the annotation files provided by the manufacturer. The median ranking value accounted for the expression value if multiple probes matched a single gene. Robust multi-array average (RMA)-normalized data were log2-transformed for further analysis.

Normalized RNA-sequencing data as transcripts per million (TPM) and the associated clinical information of the PAAD samples were downloaded from The Cancer Genome Atlas (TCGA) dataset (https://portal.gdc.cancer.gov/; ≤May 20, 2019). They included 185 cases, 182 samples, and four normal tissue samples. Eight cases without corresponding tumor samples, one case missing pathological information, fives cases with a pathological diagnosis of colloid (mucinous non-cystic) carcinoma or undifferentiated carcinoma, six cases with follow-up period ≤30 days and two samples with metastasis were eliminated. Thus, 165 cases with corresponding tumor tissues and clinical information were included in the study. Normalized gene expression data for the TCGA PAAD dataset were log2-transformed for further analysis.

### DEG Identification and Integrated Microarray Dataset Analysis

DEGs between tumor- and non-tumor tissues were identified using Limma package in R. |Log2FC| > 1, *P* < 0.05, and false discovery rate (FDR) < 0.05 were set as the cutoffs for the DEGs. The robust rank aggregation (RRA) method-based R package “RobustRankAggreg” was used for the integrated analysis of the DEGs identified from the seven GEO datasets. *P* < 0.05 was considered statistically significant. GEPIA (http://gepia.cancer-pku.cn) is a newly developed interactive web server analyzing RNA sequencing expression data for 9,736 tumors and 8,587 normal tissues in the TCGA and Genotype-Tissue Expression (GTEx) projects with a standard processing pipeline (17). As there were few normal pancreatic tissues in TCGA, the expression level of a specific DEG identified by integrated analysis of the GEO datasets were validated by GEPIA using TCGA PAAD tumor data and matched data of normal tissue from TCGA and GTEx. |Log2FC| > 1 and *P* < 0.01 were considered statistically significant. Protein expression of the DEGs in pancreatic tumor and non-tumor tissues was evaluated by the human protein atlas (https://www.proteinatlas.org/) (18). Mutation data was obtained from the cBioPortal for Cancer Genomics (https://www.cbioportal.org/) (19).

### Bioinformatic DEG Analysis

GO enrichment and KEGG pathway analyses were used to explore the potential biological processes, cellular components, and molecular functions of DEGs. Significantly relevant signal pathways were identified with DAVID (https://david.ncifcrf.gov/) (20). *P* < 0.05 was considered statistically significant. The STRING database (https://string-db.org) was used to explore potential interactions between DEGs with confidence score ≥0.4 (21). The PPI network of DEGs was constructed and visualized with Cytoscape v. 3.7.1 (https://cytoscape.org/). The Cytoscape plugin cytoHubba was used to identify hub nodes by the maximal clique centrality (MCC) method. Densely connected clusters in the PPI network were identified with the Cytoscape plugin MCODE and the default parameters. GO enrichment analysis was performed on DEG clusters.

### Identification of Survival-Related DEGs and Establishment of the Prognostic Gene Signature

The TCGA PAAD dataset was used to identify DEGs associated with overall survival. The expression levels of the DEGs identified by integrated analysis of GEO datasets were analyzed with a univariate Cox proportional hazards regression model. DEGs with *P* < 0.01 were considered statistically significant and included in subsequent analyses. Lasso-penalized Cox regression analysis was performed to further reduce the number of DEGs in the selected panel with best predictive performance using 10-fold cross validation based on glmnet package in R. A prognostic gene signature of pancreatic cancer patients was constructed based on a linear combination of the regression coefficients (β) derived from the Lasso Cox regression model multiplied with its mRNA expression level. Patients were divided into high- and low-risk groups based on the optimal cutoff of the prognostic gene signature determined using X-Tile software (22). Kaplan-Meier analysis, area under the curve (AUC) of the receiver operating characteristic (ROC) curve, Harrell's concordance index, and a calibration plot comparing predicted and observed overall survival were used to evaluate the performance of the prognostic gene signature. AJCC stage performance was used as a control. The performance of the prognostic gene signature was also compared with three previously defined gene signatures (8, 23, 24). The GSE62452 and GSE57495 datasets with complete clinical information were used for external validation. Risk scores were calculated using the prognostic gene signature. Optimal cutoffs for each dataset were determined using X-Tile. Performance of the risk score at predicting overall survival was validated using the AJCC stage as control.

### Identification of Independent Prognostic Parameters of Pancreatic Cancer

To identify independent prognostic parameters and to validate the independent prognostic value of the gene signature, univariate-, and multivariate Cox regression analyses were performed in the TCGA dataset on the prognostic gene signature and clinicopathological parameters including age, sex, tumor size, tumor site, histological subtype, grade, AJCC TNM stage, residual tumor status, surgical treatment, histories of chemotherapy, histories of radiation therapy, histories of targeted molecular therapy, tobacco smoking histories, alcohol drinking histories, histories of chronic pancreatitis, diabetes, and prior malignancy. *P* < 0.05 was considered statistically significant. Parameters with *P* < 0.05 based on the univariate analysis were further included in the multivariate Cox regression analysis.

### Predictive Nomogram Construction and Validation

After testing for collinearity, all independent prognostic parameters and relevant clinical parameters were included in the construction of a prognostic nomogram via a stepwise Cox regression model to predict 1-, 2-, and 3-year overall survival of pancreatic cancer patients in the TCGA dataset. Nomogram performance in predicting overall survival was validated using AJCC stage as control. Kaplan-Meier analysis, AUC of the ROC curve, Harrell's concordance index, and a calibration plot comparing predicted and observed overall survival were used to evaluate the performance of the prognostic nomogram. Harrell's concordance index was calculated to assess nomogram discrimination using a bootstrap method with 1,000 resamples. The nomogram calibration curve was plotted to compare predicted vs. observed overall survival. Based on the total points of the nomogram, the patients were divided into three groups by optimal cutoffs determined in X-Tile. Survival curves for the high-, medium-, and low-risk groups were plotted using Kaplan-Meier analysis.

### Gene Set Enrichment and Tumor Immunity Analyses

Gene set enrichment analysis (GSEA) was performed to elucidate the molecular mechanisms of the prognostic gene signature (25). The TCGA samples were divided into high- and low-risk groups according to the optimal cutoffs determined by X-Tile. GSEA was performed in javaGSEA v. 3.0 based on the Molecular Signatures Database v. 6.2. C2 (curated gene sets), C5 (GO gene sets), and C6 (oncogenic signatures) were searched to identify enriched KEGG pathways, biological processes, cellular components, molecular functions, and dysregulated oncogenic signatures associated with poor survival of the high-risk group. |NES| > 1 and FDR < 0.05 were considered statistically significant. Stromal, immune, and estimate scores were calculated with the ESTIMATE (estimation of stromal and immune cells in malignant tumor tissues using expression data) algorithm applied to the expression data downloaded from the TCGA PAAD dataset (https://bioinformatics.mdanderson.org/public-software/estimate/) (26). The abundances of B, CD4^+^ T, CD8^+^ T, and dendritic cells; neutrophils; and macrophages were estimated using the TIMER (tumor immune estimation resource) algorithm (https://cistrome.shinyapps.io/timer/) (27). Survival analysis of immune cell infiltration and correlation of gene expression with immune cell infiltration level in pancreatic cancer were evaluated with TIMER.

### Statistical Analysis

Statistical analysis was performed in R v. 3.4.3 and GraphPad Prism v. 8.01 (GraphPad Software, La Jolla, CA, USA). Categorical variables were analyzed by the χ^2^ or Fisher's exact test. Continuous variables were analyzed using Student's *t*-test for paired samples. Multiple groups of continuous variables were analyzed by one-way ANOVA. Univariate- and multivariate Cox regression analyses were performed to evaluate survival. The hazard ratio (HR) and 95% confidence interval (CI) were calculated to identify genes associated with overall survival. Unless otherwise stipulated, *P* < 0.05 was considered statistically significant.

## Results

### Identification of DEGs

**Figure 1:**
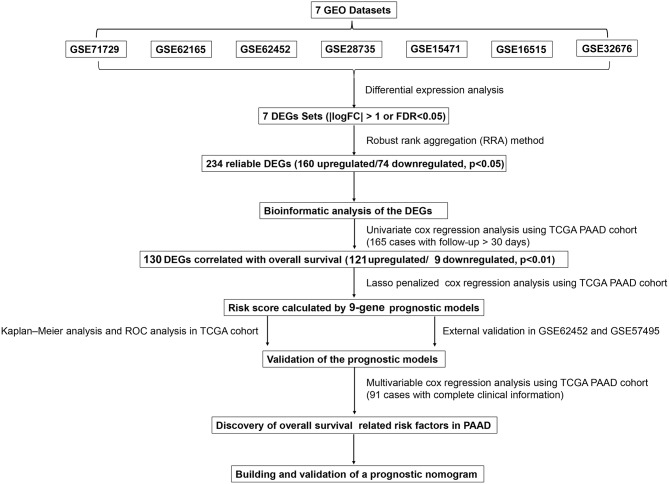
Flowchart presenting the process of establishing the gene signature and prognostic nomogram of pancreatic cancer in this study.

**Figure 2:**
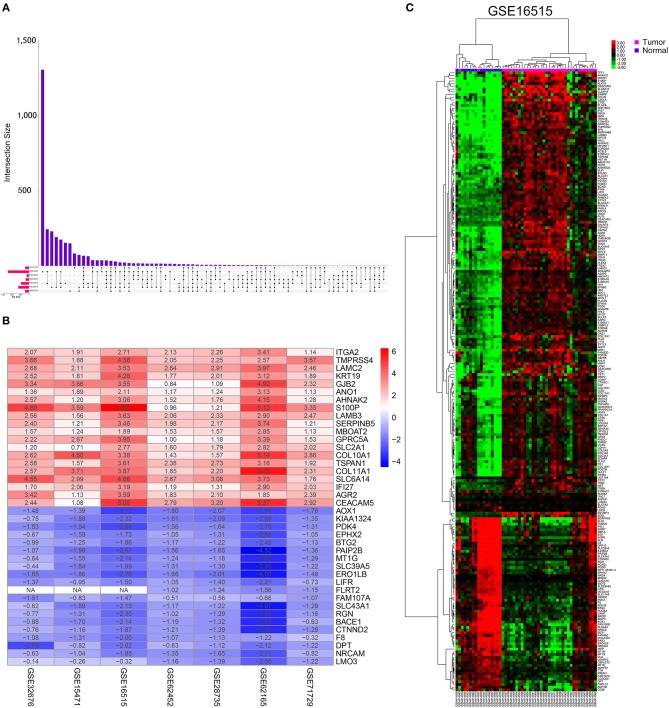
Identification of DEGs in pancreatic cancer between tumor and normal tissues. **(A)** UpSet Venn diagrams of the DEGs identified in seven GEO datasets. **(B)** The heat map of top 20 up-regulated and down-regulated DEGs identified by integrated analysis of the GEO datasets. The up-regulated DEGs are showed in red while the down-regulated DEGs are showed in blue. The value in each column represents the value of Log_2_FC. **(C)** Representative heatmap of the DEGs after integrated analysis in GSE16515 shows that the 234 DEGs can effectively distinguish tumors from non-tumor tissues.

This study was conducted according to the flow chart shown in Figure 1. Details of the GEO datasets in this study are shown in Table 1. Seven sets of DEGs (GSE71729, GSE62165, GSE62452, GSE28735, GSE15471, GSE16515, and GSE32676) comprised of 453, 2,449, 285, 395, 948, 1,238, and 472 DEGs were identified between tumor and normal tissues (Figure 2A; Supplementary Figures 1A–G). A total of 234 DEGs including 160 upregulated and 74 downregulated genes were identified after integrated analysis by robust rank aggregation (RRA) method (Supplementary Table 1). The top 20 up- and downregulated DEGs identified by integrated analysis of microarrays are shown in Figure 2B. Hierarchical clustering analysis revealed differences in DEG expression pattern between tumor and normal tissues, which could distinguish tumor from non-tumor tissues (Figure 2C; Supplementary Figures 2A–F).

**Table 1:** Details of the GEO datasets included in this study.

**Datasets**	**References**	**Platform**	**Sample size (tumor/control)**	**Application**
GSE71729	Moffitt et al. (9)	Agilent-014850 whole human genome microarray 4x44K G4112F (Gene Symbol Version; updated July, 2014)	357 (145/46)	Identification of DEGs
GSE62165	Janky et al. (10)	[HG-U219] affymetrix human genome U219 array	131 (118/13)	Identification of DEGs
GSE62452	Yang et al. (11)	[HuGene-1_0-st] affymetrix human gene 1.0 ST array [transcript (gene) version]	130 (69/61)	Identification of DEGs and validation
GSE28735	Zhang et al. (12)	[HuGene-1_0-st] affymetrix human gene 1.0 ST array [transcript (gene) version]	90 (45/45)	Identification of DEGs
GSE15471	Badea et al. (13)	[HG-U133_Plus_2] affymetrix human genome U133 Plus 2.0 array	78 (39/39)	Identification of DEGs
GSE16515	Pei et al. (14)	[HG-U133_Plus_2] affymetrix human genome U133 Plus 2.0 array	52 (36/16)	Identification of DEGs
GSE32676	Donahue et al. (15)	[HG-U133_Plus_2] affymetrix human genome U133 Plus 2.0 array	32 (25/7)	Identification of DEGs
GSE57495	Chen et al. (16)	Rosetta/Merck human RSTA custom affymetrix 2.0 microarray	63(63/0)	Validation

### Functional Enrichment and PPI Network Analysis of the DEGs

**Figure 3:**
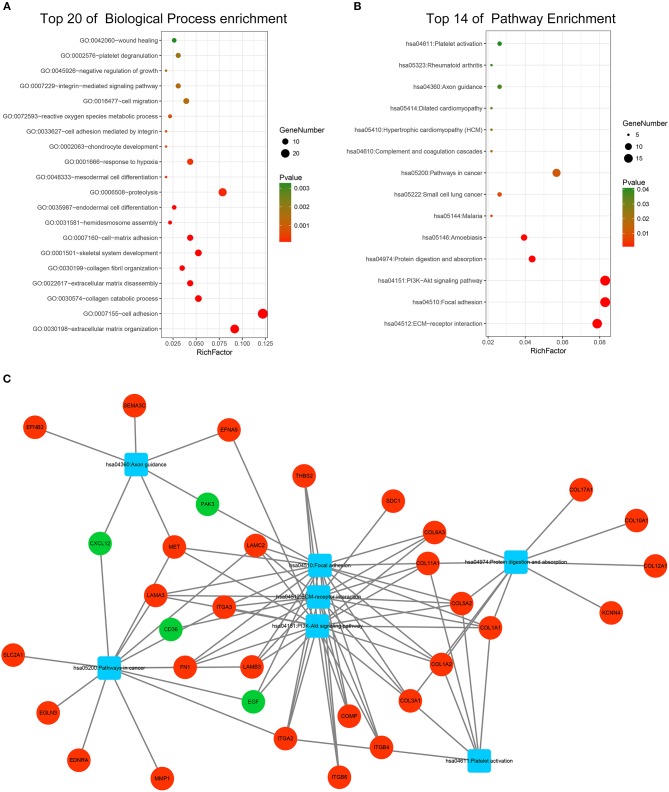
Functional enrichment analysis of the DEGs. **(A)** Top 20 enriched biological processes of the DEGs. **(B)** Top 14 enriched KEGG pathways of the DEGs. **(C)** Visualization of enriched cancer related pathways and their corresponding DEGs. Up-regulated DEGs are represented in red while down-regulated DEGs are represented in green. Pathways are represented in blue.

GO and KEGG pathway enrichment analyses were applied to discover the functions of the DEGs (Supplementary Table 2). The DEGs were significantly enriched in biological processes related to interactions between the extracellular matrix and cellular migration. This finding is consistent with the highly invasive and metastatic nature of pancreatic cancer (Figure 3A). Loss of adhesion and dissociation from *in situ* of tumor cells is the first step in invasion and metastasis. Significantly enriched biological processes included extracellular matrix organization, cell adhesion, collagen catabolic process, extracellular matrix disassembly, hemidesmosome assembly, proteolysis, and cell migration. Enrichment analyses of the cellular compartment and molecular functions are shown in Supplementary Figures 3A,B. KEGG pathway analysis revealed that the DEGs participated in PI3K-Akt signaling pathway, pathways in cancer and pathways related to cellular dissociation from *in situ*, including ECM-receptor interaction, focal adhesion, and protein digestion and absorption (Figure 3B). Furthermore, the DEGs participated in the axon guidance pathway indicating their involvement in the neurological invasion of pancreatic cancer. The interactive network of cancer-related pathways and corresponding DEGs were visualized to elucidate their associations (Figure 3C).

**Figure 4:**
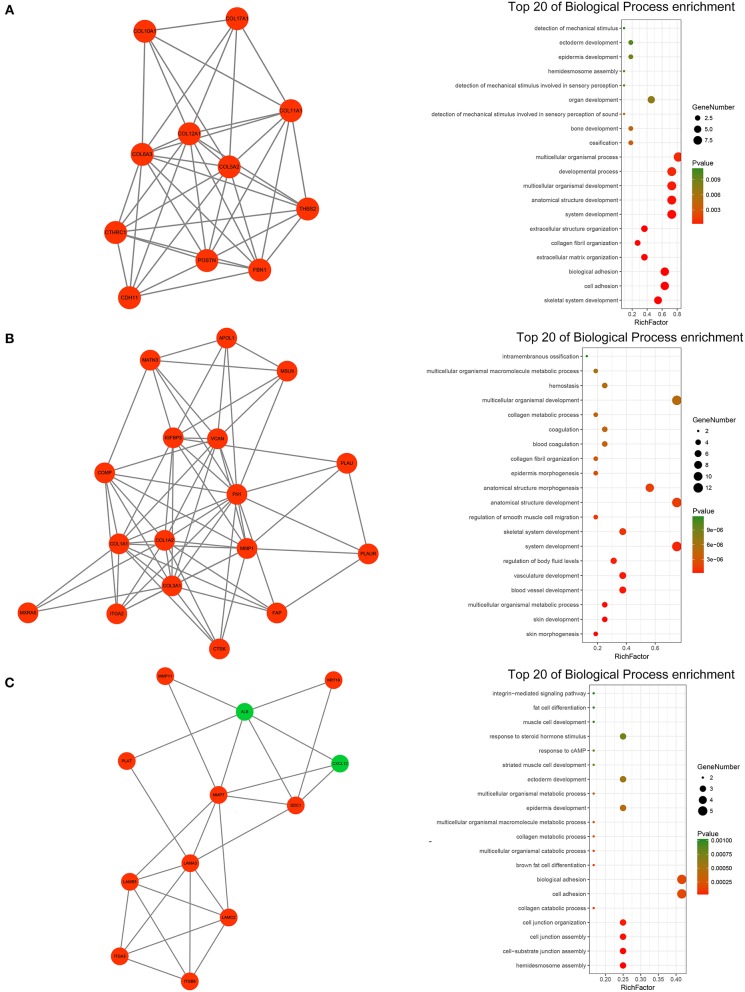
PPI network analysis of the DEGs. **(A)** Clustering module 1 with a score of 8.400 and its top 20 most enriched biological processes. **(B)** Clustering module 2 with a score of 8.125 and its top 20 most enriched biological processes. **(C)** Clustering module 3 with a score of 4.727 and its top 20 most enriched biological processes.

A PPI network of DEGs that included 186 nodes and 691 interactions was constructed to identify gene interactions. Node degree and betweenness were calculated by the MCC method to obtain hub nodes. The top 25 candidate hub genes were identified which may play a central role in this network (Supplementary Figure 3C). Module analysis identified significant clustering modules in the PPI network. The three highest-scoring clustering modules were obtained (Figures 4A–C). Each hub gene was found in ≥1 module. Thus, the three clustering modules may represent key biological roles of the PPI network. Function enrichment analysis revealed that Module 1 with a score of 8.400 was associated with cell adhesion and extracellular matrix organization. Module 2 with a score of 8.125 was correlated with blood vessel and smooth muscle development, indicating its involvement in tumor-related angiogenesis. Module 3 had a score of 4.727 and was related to cell adhesion and junction assembly. The PPI network analysis showed that the DEGs participated in pancreatic cancer progression especially in terms of invasion and metastasis.

### Identification of Survival-Related DEGs and Establishment of the Nine-Gene Prognostic Signature

One hundred sixty-five patients from the TCGA PAAD dataset with a follow-up period >30 d were included in subsequent survival analyses. The baseline characteristics of these patients are listed in Table 2. A total of 130 DEGs were identified to be significantly associated with overall survival based on the univariate Cox regression model (*P* < 0.01, Supplementary Table 3). A prognostic signature comprising nine genes, including mucolipin-3(MCOLN3), solute carrier family 25 member 45 (SLC25A45), collagen alpha-1 (XVII) chain (COL17A1), a centrosomal protein of 55 kDa (CEP55), kallikrein-10 (KLK10), hepatocyte growth factor receptor (MET), integrin beta-6 (ITGB6), ankyrin repeat domain-containing protein 22 (ANKRD22), and aryl hydrocarbon receptor nuclear translocator-like protein 2 (ARNTL2), was developed by Lasso-penalized Cox analysis (Supplementary Figure 4; Supplementary Table 4). The downregulated MCOLN3 and SLC25A45 with HR < 1 were considered as tumor suppressors, whereas the upregulated COL17A1, CEP55, KLK10, MET, ITGB6, ANKRD22, and ARNTL2 with HR > 1 were regarded as oncogenes. The risk score was calculated as follows:

(1) $$\begin{array}{l} \left[(-0.00758)\;\times \text{ Expression value of MCOLN3}\right]\\ \;+\;[(-0.03974)\;\times \text{ Expression value of SLC25A45}]\\ \;+\;[0.00658\;\times \text{ Expression value of COL17A1}]\\ \;+\;[0.11878^{*}\text{Expression value of CEP55}]\\ \;+\;[0.02763\;\times \text{ Expression value of KLK10}]\\ \;+\;[0.12604\;\times \text{ Expression value of MET}]\\ \;+\;[0.04788\;\times \text{ Expression value of ITGB6}]\\ \;+\;[0.00409\;\times \text{ Expression value of ANKRD22}]\\ \;+\;[0.09912\;\times \text{ Expression value of ARNTL2}] \end{array}$$

**Table 2:** Clinical features of pancreatic cancer patients in the TCGA Dataset.

**Clinical features**	**Mean + SD**
Follow-up time (day)	591.02 ± 479.02
Age	64.53 ± 10.84
Size (cm)	3.91 ± 1.70
	N (%)
Survival status	
Alive	76 (46.06%)
Dead	89 (53.94%)
Sex	
Male	90 (54.55%)
Female	75 (45.45%)
Site	
Head of pancreas	128 (77.58%)
Body of pancreas	14 (8.48%)
Tail of pancreas	12 (7.27%)
Others	11 (6.67%)
Subtype	
Pancreas-adenocarcinoma ductal type	140 (84.85%)
Pancreas-adenocarcinoma-other subtype	25 (15.15%)
Grade	
G1	27 (16.36%)
G2	89 (53.94%)
G3	47 (28.48%)
G4	1 (0.61%)
Not available	1 (0.61%)
T	
T1	6 (3.64%)
T2	20 (12.12%)
T3	134 (81.21%)
T4	3 (1.82%)
Not available	2 (1.21%)
N	
N0	45 (27.27%)
N1	116 (70.30%)
Not available	4 (2.42%)
M	
M0	74 (44.85%)
M1	4 (2.42%)
Mx	87 (52.73%)
AJCC stage	
I	1 (0.61%)
IA	4 (2.42%)
IB	13 (7.88%)
IIA	26 (15.76%)
IIB	112 (67.88%)
III	3 (1.82%)
IV	4 (2.42%)
Not available	2 (1.21%)
Residual tumor	
R0	96 (58.18%)
R1	51 (30.91%)
R2	5 (3.03%)
Not available	13 (7.88%)
Initial pathologic diagnosis method	
Tumor resection	99 (60.00%)
Tissue biopsy	35 (21.21%)
Cytology (e.g., Peritoneal or pleural fluid)	22 (13.33%)
Fine needle aspiration biopsy	4 (2.42%)
Not available	5 (3.03%)
Surgical treatment	
Whipple	130 (78.79%)
Distal pancreatectomy	22 (13.33%)
Distal pancreatectomy and laporoscopy followed by Hand-assisted and splenectomy	1 (0.61%)
Subtotal pancreatectomy	2 (1.21%)
Subtotal pancreatectomy and splenectomy and cholecystectomy	1 (0.61%)
Near total pancreatomy with splenectomy, duodenum perserving	1 (0.61%)
Radical pancreaticoduodenectomy	3 (1.82%)
Total pancreatectomy	2 (1.21%)
Endoscopic retrograde cholangiopancreaticography	1 (0.61%)
Not available	2 (1.21%)
History of neoadjuvant treatment	
No	164 (99.39%)
Yes	1 (0.61%)
History of chemotherapy	
No	51 (30.91%)
Yes	114 (69.09%)
History of radiation therapy	
No	94 (56.97%)
Yes	45 (27.27%)
Not available	26 (15.76%)
History of targeted molecular therapy	
No	41 (24.85%)
Yes	112 (67.88%)
Not available	12 (7.27%)
Tobacco smoking history	
Lifelong non-smoker	60 (36.36%)
Current smoker	18 (10.91%)
Current reformed smoker for >15 years	27 (16.36%)
Current reformed smoker for ≤15 years	22 (13.33%)
Current reformed smoker, duration not specified	7 (4.24%)
Not available	31 (18.79%)
Alcohol drinking history	
No	61 (36.97%)
Yes	92 (55.76%)
Not available	12 (7.27%)
History of chronic pancreatitis	
No	119 (72.12%)
Yes	13 (7.88%)
Not available	33 (20.00%)
History of diabetes	
No	102 (61.82%)
Yes	34 (20.61%)
Not available	29 (17.58%)
History of prior malignancy	
No	148 (89.70%)
Yes	17 (10.30)

**Figure 5:**
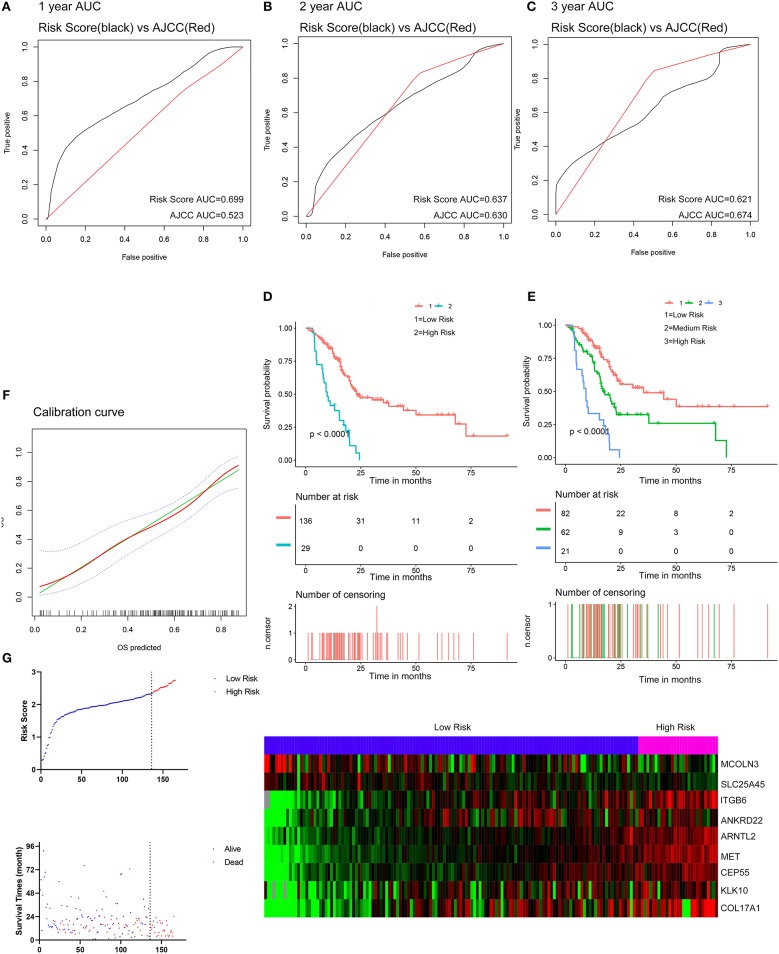
Evaluation of the performance of the nine gene signature in TCGA PAAD dataset. **(A–C)** Show the ROC curves for 1-, 2-, and 3-year overall survival predictions for the nine gene signature in compare with AJCC stage. **(D,E)** Show the Kaplan-Meier survival curves of the nine gene signature. Patients from the TCGA dataset are stratified into two or three groups according to the optimal cut-off values for the risk scores calculated by X-Tile. **(F)** The calibration plot for internal validation of the nine gene signature. The Y axis represents the actual overall survival while the X axis represents the predicted overall survival. **(G)** Distribution of the risk score, the associated survival data and the mRNA expression heat map in the TCGA dataset.

The optimal cutoff values for the risk scores were calculated with X-Tile software. Patients from the TCGA dataset were stratified into two (cutoff value = 2.33) or three (cutoff values = 2.01 and 2.45) groups. The Kaplan-Meier survival curves revealed significantly favorable overall survival in all groups with lower risk scores (*P* < 0.0001) (Figures 5D,E). Time-dependent ROC and C-index were applied to determine the prognostic values of the nine gene risk scores compared with the AJCC stage (Figures 5A–C). The AUCs for 1-, 2-, and 3-year overall survival predictions for the risk scores were 0.699, 0.637, and 0.621, respectively. The AUCs for 1-, 2-, and 3-year overall survival predictions for the AJCC stage were 0.523, 0.630, and 0.674, respectively. The C-index of the risk score was 0.673 (95% CI; 0.614–0.732), while that of the AJCC stage was 0.562 (95% CI; 0.507–0.618). The calibration curves for the risk score revealed that the predicted overall survival accorded with the observed overall survival (Figure 5F). The performance of the risk score was also compared with three previously defined gene signatures. The risk score had the highest C-index (0.673 vs. 0.625, 0.612, and 0.544) indicating a superior prognostic value (Supplementary Figures 5A–C). The performance of the risk score was further explored in different subgroups of patients (Supplementary Figure 6). The risk score performed well in predicting overall survival of patients in subgroup of stage I and II and subgroups without the history of chemotherapy, molecular targeted therapy, and radiation therapy, forecasting the natural course of pancreatic cancer. We further explore whether the relative treatment benefit varies according to the values of the risk score. Kaplan-Meier analyses reveal that patients with higher risk score (top 50%) have better response to chemotherapy, molecular targeted therapy, and radiation therapy than patients with lower risk score (bottom 50%; Supplementary Figure 7). In general, the nine-gene signature performed well at predicting overall survival of pancreatic cancer.

### External Validation of the Prognostic Performance of the Nine Gene Signature

**Figure 6:**
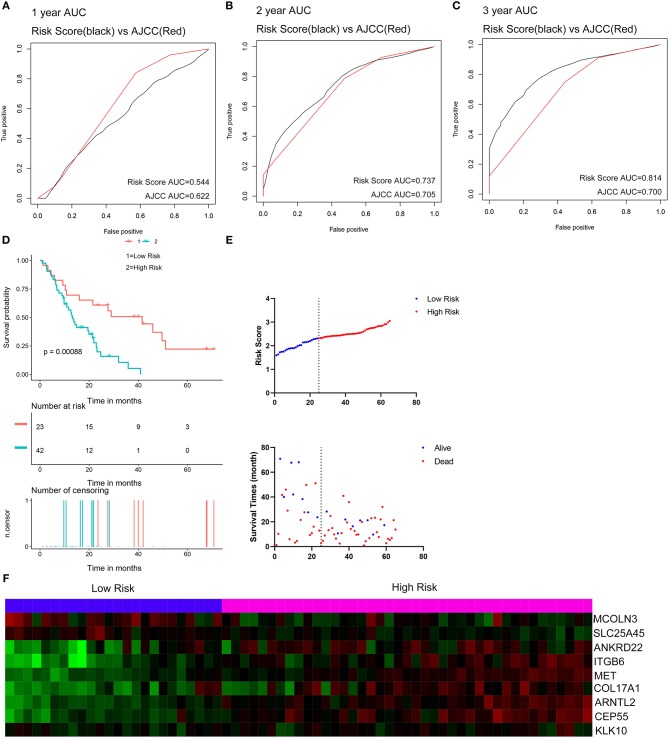
External validation of the nine gene signature in GSE62452 dataset. **(A–C)** Show the ROC curves for 1-, 2-, and 3-year overall survival predictions for the nine gene signature in compare with AJCC stage. **(D)** shows the Kaplan-Meier survival curves of the nine gene signature. Patients from the GSE62452 dataset are stratified into two groups according to the optimal cut-off values for the risk scores calculated by X-Tile. **(E,F)** Distribution of the risk score and the associated survival data and mRNA expression heat map in GSE62452 dataset.

Two external datasets GSE62452 and GSE57495 were used to validate the prediction performance of the nine-gene prognostic signature (Figure 6; Supplementary Figure 8). Risk scores were calculated with the same formula for each patient. Patients were divided into high- and low-risk groups according to the optimal cutoffs determined for each dataset. The Kaplan-Meier survival curves revealed significant difference in overall survival between groups in both datasets. High-risk groups had markedly poorer outcomes than low-risk groups (Figure 6D and Supplementary Figure 8D). Prognostic power was then assessed by time-dependent ROC and C-index. In both datasets, the nine-gene signature had a comparable or superior performance to that of the AJCC stage. In GSE62452, the AUCs for 1-, 2-, and 3-year overall survival predictions for the risk scores were 0.544, 0.737, and 0.814, respectively. The AUCs for 1-, 2-, and 3-year overall survival predictions for the AJCC stage were 0.622, 0.705, and 0.700, respectively. The C-index of the risk score was 0.582 (95% CI; 0.482–0.681), while that for the AJCC stage was 0.603 (95% CI; 0.519–0.687) (Figures 6A–C). In GSE57495, the AUCs for 1-, 2-, and 3-year overall survival predictions for the risk score were 0.658, 0.612, and 0.670, respectively. The AUCs for 1-, 2-, and 3-year overall survival predictions for the AJCC stage were 0.595, 0.660, and 0.654, respectively. The C-index of the risk score was 0.612 (95% CI; 0.517–0.707), while that for the AJCC stage was 0.600 (95% CI; 0.516–0.683) (Supplementary Figures 8A–C). External validation indicated that the nine-gene signature performed well at predicting overall survival in pancreatic cancer patients.

### Validation of Expression and Alteration of the Nine Genes

**Figure 7:**
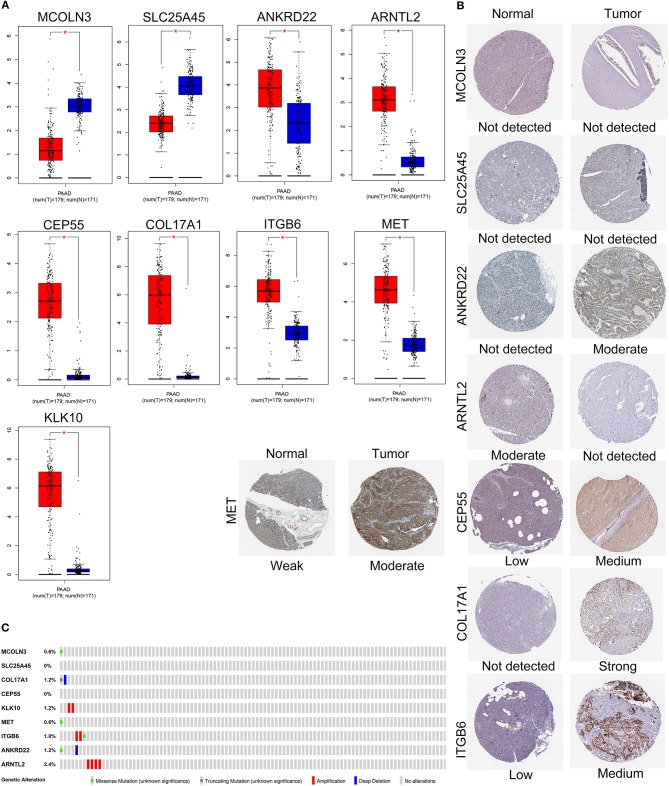
Validation of expression and alteration of the nine genes in pancreatic cancer. **(A)** The mRNA expression levels in TCGA pancreatic cancer tumor tissue and matching normal tissue from data of TCGA and GTEx. Data was obtained from the GEPIA (http://gepia.cancer-pku.cn/). **(B)** The representative protein expression of the nine genes in pancreatic cancer tumor tissue and normal tissue. Data was obtained from the human protein atlas (https://www.proteinatlas.org/). **(C)** Genetic alterations of the nine genes in pancreatic cancer. Data was obtained from the cBioportal (https://www.cbioportal.org/).

The expression levels of the nine genes were validated using GEPIA. The mRNA expression levels of COL17A1, CEP55, KLK10, MET, ITGB6, ANKRD22, and ARNTL2 were significantly increased in PAAD tumor tissue. In contrast, MCOLN3 and SLC25A45 were significantly decreased in compare with non-tumor tissues (Figure 7A). Human protein atlas database was used to explore protein expression levels. Typical IHC of eight genes (except KLK10, not included in the database) in tumor and normal pancreatic tissues are shown in Figure 7B. (Images are available from v18.proteinatlas.org). Of the 165 PAAD patients included in the current study, 15 (9%) presented with alterations in the nine genes. Amplification was the most common type of mutation in the upregulated genes (Figure 7C).

### Evaluation of Prognostic Factors in PAAD

Ninety-one patients from the TCGA PAAD dataset for which complete clinical information was provided, including age, sex, tumor size, tumor site, histological subtype, grade, AJCC TNM stage, residual tumor status, surgical treatment, histories of chemotherapy, histories of radiation therapy, histories of targeted molecular therapy, tobacco smoking histories, alcohol drinking histories, histories of chronic pancreatitis, diabetes, and prior malignancy, were included in the analysis (Table 3). Reasons of exclusion for each case was listed in the Supplementary Table 5. Prognostic factors of overall survival for pancreatic cancer were identified using univariate- and multivariate cox regression analyses. The unadjusted univariate analysis revealed that risk score (*P* = 0.0005), tumor size (*P* = 0.0235), tumor site (*P* = 0.0225; body and tail of pancreas vs. head), histological subtype (*P* = 0.0215; other subtypes of adenocarcinoma vs. ductal type), T stage (*P* = 0.0129; T3 and T4 vs. T1 and T2), N stage (*P* = 0.0049; N1 vs. N0), AJCC stage (*P* = 0.0224; IIB vs. I; *P* = 0.0108; III and IV vs. I), residual tumor (*P* = 0.0320; R1 vs. R0), history of chemotherapy (*P* = 0.0202), history of radiation therapy (*P* = 0.0228), and history of targeted molecular therapy (*P* < 0.0001) were significantly correlated with overall survival of pancreatic cancer (Table 4). Multivariate analysis revealed that risk score, tumor size, and history of targeted molecular therapy were independent risk factors of overall survival (*P* < 0.05; Table 5).

**Table 3:** Baseline characteristics of patients included for univariate and multivariate Cox regression analysis.

**Clinical features**	**Mean + SD**
Risk score	2.06 ± 0.44
Age	63.59 ± 11.31
Follow-up time (day)	528.46 ± 371.92
Size (cm)	3.77 ± 1.44
	N (%)
Survival status	
Alive	35 (38.46%)
Dead	56 (61.54%)
Sex	
Male	52 (57.14%)
Female	39 (42.86%)
Site	
Head of pancreas	74 (81.32%)
Body of pancreas	5 (5.49%)
Tail of Pancreas	9 (9.89%)
Others	3 (3.30%)
Subtype	
Pancreas-adenocarcinoma ductal type	79 (86.81%)
Pancreas-adenocarcinoma-other subtype	12 (13.19%)
Grade	
G1	9 (9.89%)
G2	51 (56.04%)
G3	30 (32.97%)
G4	1 (1.10%)
T	
T1	4 (4.40%)
T2	11 (12.09%)
T3	75 (82.42%)
T4	1 (1.10%)
N	
N0	26 (28.57%)
N1	65 (71.43%)
M	
M0	54 (59.34%)
M1	1 (1.10%)
Mx	36 (39.56%)
AJCC stage	
IA	3 (3.30%)
IB	8 (8.79%)
IIA	14 (15.38%)
IIB	64 (70.33%)
III	1 (1.10%)
IV	1 (1.10%)
Residual tumor	
R0	55 (60.44%)
R1	34 (37.36%)
R2	2 (2.20%)
Initial pathologic diagnosis method	
Tumor resection	53 (58.24%)
Tissue biopsy	23 (25.27%)
Cytology (e.g., Peritoneal or pleural fluid)	13 (14.29%)
Fine needle aspiration biopsy	1 (1.10%)
Not available	1 (1.10%)
Surgical treatment	
Whipple	74 (81.32%)
Distal pancreatectomy	12 (13.19%)
Distal pancreatectomy and laporoscopy followed by Hand-assisted and splenectomy	1 (1.10%)
Subtotal pancreatectomy	1 (1.10%)
Subtotal pancreatectomy and splenectomy and cholecystectomy	1 (1.10%)
Total pancreatectomy	1 (1.10%)
Endoscopic retrograde cholangiopancreaticography	1 (1.10%)
History of neoadjuvant treatment	
No	90 (98.90%)
Yes	1 (1.10%)
History of chemotherapy	
No	25 (27.47%)
Yes	66 (72.53%)
History of radiation therapy	
No	67 (73.63%)
Yes	24 (26.37%)
History of targeted molecular therapy	
No	29 (31.87%)
Yes	62 (68.13%)
Tobacco smoking history	
Lifelong non-smoker	34 (37.36%)
Current smoker	16 (17.58%)
Current reformed smoker for >15 years	21 (23.08%)
Current reformed smoker for ≤15 years	15 (16.48%)
Current reformed smoker, duration not specified	5 (5.49%)
Alcohol drinking history	
No	25 (27.47%)
Yes	66 (72.53%)
History of chronic pancreatitis	
No	80 (87.91%)
Yes	11 (12.09%)
History of diabetes	
No	67 (73.63%)
Yes	24 (26.37%)
History of prior malignancy	
No	82 (90.11%)
Yes	9 (9.89%)

**Table 4:** Unadjusted univariate analysis.

**Exposure**	**Statistics**	**Overall survival**
Risk score	2.06 ± 0.44	5.45 (2.09, 14.17) 0.0005
Risk score tertile		
Low	30 (32.97%)	1
Middle	30 (32.97%)	1.56 (0.79, 3.08) 0.1991
High	31 (34.07%)	2.17 (1.12, 4.19) 0.0218
Age	63.59 ± 11.31	1.02 (1.00, 1.05) 0.0663
Age tertile		
Low	30 (32.97%)	1
Middle	29 (31.87%)	1.07 (0.53, 2.16) 0.8510
High	32 (35.16%)	1.68 (0.88, 3.22) 0.1155
Sex		
Male	52 (57.14%)	1
Female	39 (42.86%)	1.06 (0.62, 1.79) 0.8367
Size(cm)	3.77 ± 1.44	1.24 (1.03, 1.49) 0.0235
Size(cm) tertile		
Low	26 (28.57%)	1
Middle	25 (27.47%)	0.91 (0.43, 1.92) 0.7992
High	40 (43.96%)	1.33 (0.72, 2.46) 0.3660
Site		
Head of pancreas	74 (81.32%)	1
Body and tail of pancreas and others	17 (18.68%)	0.37 (0.16, 0.87) 0.0225
Subtype		
Pancreas-adenocarcinoma ductal type	79 (86.81%)	1
Pancreas-adenocarcinoma- other subtype	12 (13.19%)	0.30 (0.11, 0.84) 0.0215
Grade		
G1 and G2	60 (65.93%)	1
G3 and G4	31 (34.07%)	1.57 (0.92, 2.68) 0.0975
T		
T1 and T2	15 (16.48%)	1
T3 and T4	76 (83.52%)	3.24 (1.28, 8.20) 0.0129
N		
N0	26 (28.57%)	1
N1	65 (71.43%)	2.69 (1.35, 5.36) 0.0049
M		
M0	54 (59.34%)	1
M1	1 (1.10%)	2.10 (0.28, 15.54) 0.4681
Mx	36 (39.56%)	0.79 (0.46, 1.36) 0.4016
AJCC stage		
I	11 (12.09%)	1
IIA	14 (15.38%)	1.33 (0.35, 4.99) 0.6770
IIB	63 (69.23%)	3.34 (1.19, 9.41) 0.0224
III and IV	3 (3.30%)	7.42 (1.59, 34.63) 0.0108
Residual tumor		
R0	55 (60.44%)	1
R1	34 (37.36%)	1.82 (1.05, 3.14) 0.0320
R2	2 (2.20%)	1.72 (0.23, 12.82) 0.5976
Surgical treatment		
Whipple	74 (81.32%)	1
Distal pancreatectomy	13 (14.29%)	0.51 (0.22, 1.20) 0.1237
Subtotal pancreatectomy	2 (2.20%)	0.00 (0.00, Inf) 0.9973
Others	2 (2.20%)	0.00 (0.00, Inf) 0.9978
History of chemotherapy		
No	25 (27.47%)	1
Yes	66 (72.53%)	0.52 (0.30, 0.90) 0.0202
History of radiation therapy		
No	67 (73.63%)	1
Yes	24 (26.37%)	0.45 (0.23, 0.89) 0.0228
History of targeted molecular therapy		
No	29 (31.87%)	1
Yes	62 (68.13%)	0.30 (0.17, 0.51) <0.0001
Tobacco smoking history		
Lifelong non-smoker	34 (37.36%)	1
Current or former smoker	57 (62.64%)	0.77 (0.45, 1.32) 0.3397
Alcohol drinking history		
No	25 (27.47%)	1
Yes	66 (72.53%)	1.27 (0.70, 2.30) 0.4252
History of chronic pancreatitis		
No	80 (87.91%)	1
Yes	11 (12.09%)	0.81 (0.37, 1.79) 0.6029
History of diabetes		
No	67 (73.63%)	1
Yes	24 (26.37%)	0.94 (0.50, 1.75) 0.8422
History of prior malignancy		
No	82 (90.11%)	1
Yes	9 (9.89%)	1.18 (0.50, 2.77) 0.7006

**Table 5:** Multivariate Cox regression analysis.

**Exposure**	**Non-adjusted**	**Adjust I**	**Adjust II**	**Adjust III**
Risk score	5.45 (2.09, 14.17) 0.0005	4.74 (1.73, 12.96) 0.0025	4.74 (1.73, 12.96) 0.0025	3.58 (1.50, 8.51) 0.0040
Age	1.02 (1.00, 1.05) 0.0663	1.02 (1.00, 1.05) 0.0872	1.03 (1.00, 1.05) 0.0536	NA
Sex				
Male	1	1	1	NA
Female	1.06 (0.62, 1.79) 0.8367	0.88 (0.51, 1.52) 0.6477	0.86 (0.50, 1.48) 0.5961	NA
Size(cm)	1.24 (1.03, 1.49) 0.0235	1.19 (0.96, 1.47) 0.1219	1.19 (0.96, 1.47) 0.1167	1.30 (1.00, 1.68) 0.0463
Site				
Head of pancreas	1	1	1	1
Body and tail of pancreas and others	0.37 (0.16, 0.87) 0.0225	0.51 (0.21, 1.23) 0.1351	0.49 (0.20, 1.19) 0.1143	0.44 (0.16, 1.15) 0.0944
Subtype				
Pancreas-adenocarcinoma ductal type	1	1	1	1
Pancreas-adenocarcinoma-other subtype	0.30 (0.11, 0.84) 0.0215	0.53 (0.18, 1.58) 0.2524	0.78 (0.26, 2.29) 0.6462	0.49 (0.16, 1.47) 0.2005
Grade				
G1 and G2	1	1	1	NA
G3 and G4	1.57 (0.92, 2.68) 0.0975	1.39 (0.80, 2.42) 0.2490	1.22 (0.70, 2.14) 0.4853	NA
T				
T1 and T2	1	1	1	1
T3 and T4	3.24 (1.28, 8.20) 0.0129	4.00 (0.53, 29.92) 0.1770	4.33 (0.58, 32.56) 0.1543	4.50 (0.60, 33.92) 0.1439
N				
N0	1	1	1	1
N1	2.69 (1.35, 5.36) 0.0049	0.89 (0.08, 10.23) 0.9268	0.48 (0.04, 5.72) 0.5603	0.21 (0.01,3.20) 0.2603
M				
M0	1	1	1	NA
M1	2.10 (0.28, 15.54) 0.4681	0.44 (0.04, 5.46) 0.5261	0.53 (0.04, 6.58) 0.6213	NA
Mx	0.79 (0.46, 1.36) 0.4016	0.74 (0.42, 1.30) 0.2906	0.81 (0.46, 1.43) 0.4621	NA
AJCC stage				
I	1	1	1	1
IIA	1.33 (0.35, 4.99) 0.6770	1.25 (0.33, 4.77) 0.7401	0.96 (0.26, 3.64) 0.9571	0.22 (0.02, 2.63) 0.2340
IIB	3.34 (1.19, 9.41) 0.0224	3.13 (1.11, 8.83) 0.0315	2.11 (0.75, 5.92) 0.1553	2.00 (0.06, 64.49) 0.6957
III and IV	7.42 (1.59, 34.63) 0.0108	8.61 (1.81, 40.88) 0.0068	4.07 (0.85, 19.52) 0.0796	1.60 (0.07, 36.94) 0.7701
Residual tumor				
R0	1	1	1	1
R1	1.82 (1.05, 3.14) 0.0320	1.63 (0.93, 2.87) 0.0895	1.66 (0.95, 2.90) 0.0754	1.25 (0.68, 2.29) 0.4658
R2	1.72 (0.23, 12.82) 0.5976	1.94 (0.24, 15.36) 0.5312	2.26 (0.28, 18.34) 0.4447	3.55 (0.43, 29.18) 0.2377
Surgical treatment				
Whipple	1	1	1	NA
Distal pancreatectomy	0.51 (0.22, 1.20) 0.1237	0.72 (0.29, 1.76) 0.4649	0.65 (0.27, 1.58) 0.3419	NA
Subtotal pancreatectomy	0.00 (0.00, Inf) 0.9973	0.00 (0.00, Inf) 0.9975	0.00 (0.00, Inf) 0.9973	NA
Others	0.00 (0.00, Inf) 0.9978	0.00 (0.00, Inf) 0.9979	0.00 (0.00, Inf) 0.9980	NA
History of chemotherapy				
No	1	1	1	1
Yes	0.52 (0.30, 0.90) 0.0202	0.21 (0.11, 0.40) <0.0001	0.20 (0.10, 0.39) <0.0001	0.85 (0.31, 2.33) 0.7553
History of radiation therapy				
No	1	1	1	1
Yes	0.45 (0.23, 0.89) 0.0228	0.53 (0.26, 1.06) 0.0729	0.54 (0.27, 1.09) 0.0866	0.83 (0.38, 1.83) 0.6512
History of targeted molecular therapy				
No	1	1	1	1
Yes	0.30 (0.17, 0.51) <0.0001	0.17 (0.09, 0.31) <0.0001	0.16 (0.08, 0.31) <0.0001	0.17 (0.06, 0.48) 0.0009
Tobacco smoking history				
Lifelong non-smoker	1	1	1	NA
Current or former smoker	0.77 (0.45, 1.32) 0.3397	0.76 (0.44, 1.31) 0.3207	0.78 (0.45, 1.34) 0.3657	NA
Alcohol drinking history				
No	1	1	1	NA
Yes	1.27 (0.70, 2.30) 0.4252	1.34 (0.70, 2.57) 0.3778	1.10 (0.58, 2.08) 0.7676	NA
History of chronic pancreatitis				
No	1	1	1	NA
Yes	0.81 (0.37, 1.79) 0.6029	1.09 (0.47, 2.52) 0.8359	0.84 (0.36, 1.97) 0.6950	NA
History of diabetes				
No	1	1	1	NA
Yes	0.94 (0.50, 1.75) 0.8422	0.92 (0.49, 1.75) 0.8053	0.85 (0.45, 1.59) 0.6058	NA
History of Prior Malignancy				
No	1	1	1	NA
Yes	1.18 (0.50, 2.77) 0.7006	1.19 (0.49, 2.90) 0.7076	1.05 (0.42, 2.59) 0.9197	NA

*Adjust I model adjust for: Age, Sex and AJCC Stage*.

*Adjust II model adjust for: Age, Sex, AJCC Stage and Risk Score*.

*Adjust III model adjust for parameters associated with overall survival based on univariate analysis*.

### Building and Validating a Predictive Nomogram

**Figure 8:**
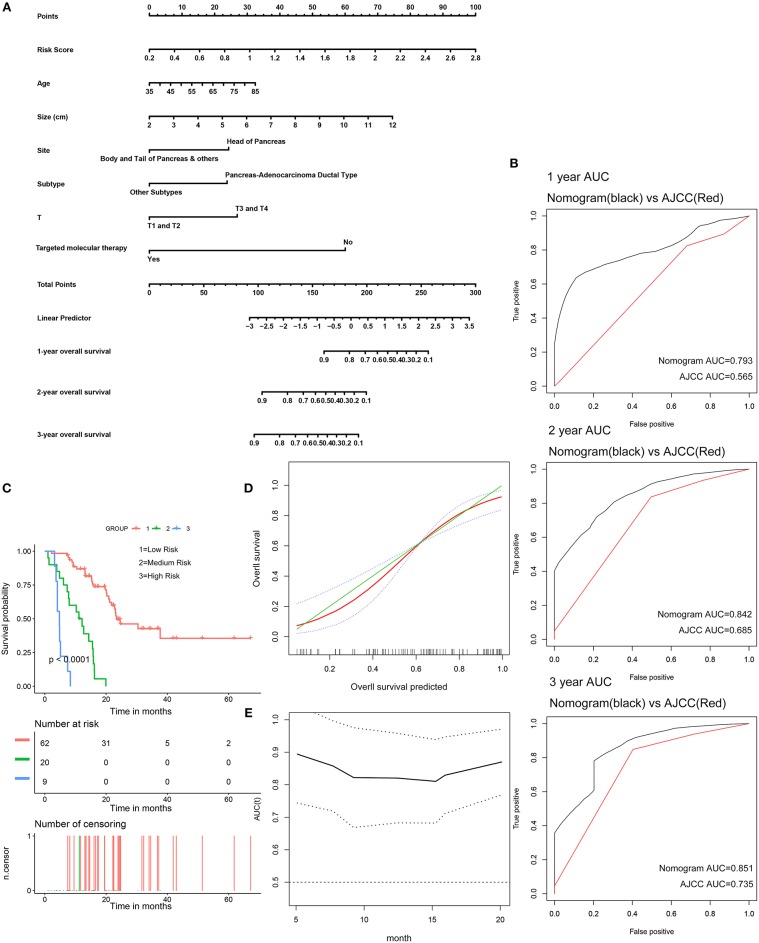
Validation of the nomogram in predicting overall survival of pancreatic cancer in the TCGA dataset. **(A)** A prognostic nomogram predicting 1-, 2-, and 3-year overall survival of pancreatic cancer. **(B)** Shows the time-dependent ROC for 1-, 2-, and 3-year overall survival predictions for the nomogram in compare with AJCC stage. **(C)** Shows the Kaplan-Meier survival curves of the nomogram. Patients from the TCGA dataset are stratified into three groups according to the optimal cutoffs for the nomogram calculated by X-Tile. **(D)** The calibration plot for internal validation of the nomogram. The Y axis represents the actual overall survival while the X axis represents the predicted overall survival. **(E)** The time dependent AUC of the nomogram in predicting overall survival of pancreatic cancer.

The 91 patients with complete clinical information from the TCGA dataset were used to establish a prognostic nomogram predicting 1-, 2-, and 3-year overall survival based on the stepwise Cox regression model (Figure 8A). Risk score, age, tumor size, tumor site, histological subtype, T stage, and histories of targeted molecular therapy were parameters included in the nomogram. The AUCs of the 1-, 2-, and 3-year overall survival predictions for the nomogram were 0.793, 0.842, and 0.851, respectively. The AUCs of the 1-, 2-, and 3-year overall survival predictions for the AJCC stage were 0.565, 0.685, and 0.735, respectively. The C-index of the risk score was 0.779 (95% CI; 0.714–0.845), while that for the AJCC stage was 0.587 (95% CI; 0.520–0.654). Thus, the nomogram was superior to the AJCC stage in terms of predicting overall survival of pancreatic cancer (Figure 8B). The patients were divided into three groups of equal size according to scoring of nomogram. The Kaplan-Meier plot effectively discriminated these groups of various risk (Figure 8C). Those with higher scores had significantly poorer overall survival (*P* < 0.0001). Calibration plots showed that the nomogram performed well at predicting overall survival in pancreatic cancer patients (Figure 8D). When the predicted overall survival was >80 or <60%, the nomogram may underestimate the mortality.

### Gene Set Enrichment Analysis (GSEA)

**Figure 9:**
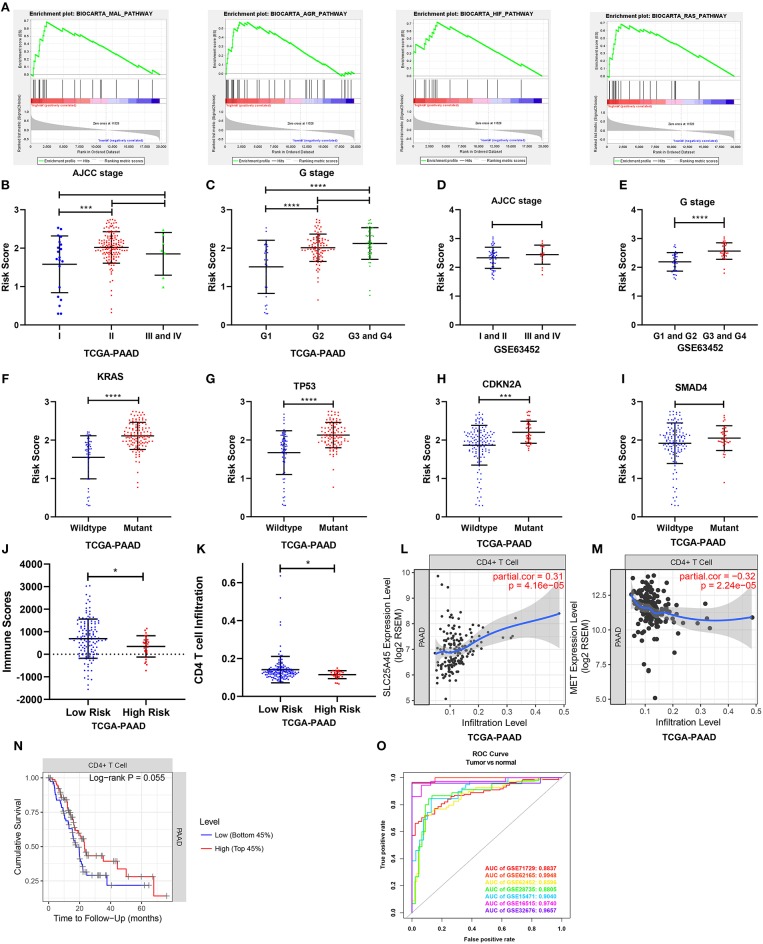
Gene set enrichment analysis and the clinical- and tumor immunity relevance of the nine-gene signature. **(A)** Top 4 oncological signatures significantly enriched in the high-risk group identified by gene set enrichment analysis. **(B,D)** Show the distribution of the risk score in different AJCC stage in TCGA and GSE63452 datasets. **(C,E)** Show the distribution of the risk score in different grade in TCGA and GSE63452 datasets. **(F–I)** Show the distribution of the risk score in different mutation status of KRAS, TP53, CDKN2A, and SMAD4 in TCGA dataset. **(J)** Show the distribution of the immune score in high risk and low risk group in TCGA datasets. Immune scores were calculated with the ESTIMATE algorithm (https://bioinformatics.mdanderson.org/public-software/estimate/). **(K)** Shows the CD4 T cell infiltration level in high risk and low risk group in TCGA datasets. The abundances of CD4^+^ T cells were estimated using the TIMER algorithm (https://cistrome.shinyapps.io/timer/). **(L,M)** Show the correlation of SLC25A45 and MET expression with immune infiltration level in pancreatic cancer. **(O)** The ROC curves of the risk score differentiating pancreatic cancer from normal tissues in the seven GEO datasets. *P < 0.05, ***P < 0.001, ****P < 0.0001.

To elucidate the molecular mechanism of the nine-gene signature, 165 patients from the TCGA PAAD dataset were divided into high- and low-risk groups according to the optimal cutoff for the nine-gene risk score determined by X-tile software. GSEA compared the high- and low-risk groups. In the former, 23 oncological signatures were significantly enriched including the MAL, AGR, HIF, RAS, ECM, ATRBRCA, PTC1, and other pathways (Figure 9A). KEGG pathways enriched in the high-risk group included regulation of the actin cytoskeleton, ubiquitin-mediated proteolysis, axon guidance, focal adhesion, and tight junction. These enriched KEGG pathways revealed that molecular alteration in the high-risk group was closely associated with the malignant properties of pancreatic cancer, especially invasion and metastasis. Results of the GSEA are shown in Supplementary Table 6.

### Clinical- and Tumor Immunity Relevance of the Nine-Gene Signature

Relationships between the nine-gene signature and the clinical characteristics of pancreatic cancer (including AJCC stage, grade, metastasis, and key gene mutation state) were analyzed in datasets providing necessary clinical information. In terms of AJCC stage, stage II patients had higher risk scores than stage I patients in the TCGA dataset. But the risk scores for the stage III and IV patients were not higher than those for the stage II patients (Figure 9B). The risk scores were also comparable across different AJCC stages in GSE63452, GSE62165, and GSE57495 (Figure 9D; Supplementary Figures 9B,C). In terms of grade, patients of G2, G3, and G4 had higher risk scores than G1 (Figure 9C), which was consistent with the finding from GSE63452 (Figure 9E). Moreover, data from GSE71729 revealed that the risk scores for the metastases were higher than the primary tumors (Supplementary Figure 9A). In terms of mutation, risk scores were identified to be highly associated with mutation state of key genes. The risk scores for the KRAS, TP53, and CDKN2A mutant groups were significantly higher than those for the wild type (Figures 9F–H). The risk score in the SMAD4 mutant group was non-significantly higher than that for the wild type (Figure 9I). Finally, the performance of the nine-gene signature at differentiating pancreatic cancer from normal tissue were explored across all the seven GEO datasets (Figure 9O). Tumor tissues could be effectively identified based on the risk score.

To investigate tumor immunity relevance of the nine-gene signature, the associations of the gene signature with tumor purity and the presence of infiltrating stromal/immune cells in tumor tissues were evaluated. Stromal-, immune-, and estimate scores were calculated by applying the ESTIMATE algorithm to the expression data downloaded from the TCGA PAAD dataset. The stromal- and estimate scores were comparable between the high- and low-risk groups. However, the immune score was significantly lower in the high-risk group, indicating fewer infiltration of immune cells in the tumor tissue (*P* < 0.05; Figure 9J). The abundances of B, CD4^+^ T, CD8^+^ T, and dendritic cells; neutrophils; and macrophages were further estimated using the TIMER algorithm. The high-risk group was associated with relatively lower levels of CD4^+^ T cell infiltration (Figure 9K). Consistently, the downregulated SLC25A45 was positively correlated with CD4^+^ T cell infiltration level, whereas the upregulated MET was negatively associated with it (*P* < 0.0001) (Figures 9L,M). In addition, a lower level of CD4^+^ T cell infiltration was associated with poor survival (*P* = 0.055; Figure 9N).

## Discussion

Pancreatic cancer is highly malignant with very poor prognosis. The 5-year survival rate is about 5% (1). Accurate prediction of prognosis can identify patients benefiting from more radical treatment, including neoadjuvant therapy, more intensive surgery, chemotherapy, radiation therapy, targeted molecular therapy, and immunotherapy. Therefore, treatments can be tailored to individual patients to improve prognosis. Traditional clinicopathological parameters have been applied to reflect and prognosticate disease progression. AJCC staging is currently the most effective tool for prognostic prediction of pancreatic cancer. Besides, molecular prognostic markers may be used as a beneficial supplement to AJCC staging to further improve the accuracy of prognosis prediction. Molecular prognostic markers which can be quantified by standardized detection procedures vary with tumor progression and may dynamically reflect the prognosis of patients. Moreover, they may also play important roles in progression of pancreatic cancer and serve as new therapeutic targets. By combining with the detection of tumor-associated exosomes and circulating tumor cells (CTC), real-time detection of disease recurrence and treatment response in patients after surgical resection may be achieved. Molecular prognostic markers may also have potential value in early diagnosis of the highly heterogeneous pancreatic cancer, progression of which involves a complex network of multiple signaling pathways. To overcome the hinder of heterogeneity, a panel of molecular markers may be more accurate in reflecting pancreatic cancer prognosis than a single one. Nomograms are widely used in clinical oncology to evaluate prognosis (28). They can integrate several prognostic determinants including molecular and clinicopathological parameters. The numerical probabilities of clinical events can be calculated and visualized with relatively simple output. Compared to conventional staging, nomograms may effectively improve the prediction of prognosis, which is beneficial for the clinical decision-making and personalized treatment.

In the current study, we identified 234 reliable DEGs of pancreatic cancer by integrative analysis of multiple datasets. Functional enrichment analysis revealed that the DEGs were closely related to invasion and metastasis of pancreatic cancer. PI3K-Akt was the most enriched signaling pathway. Survival analysis revealed 130 DEGs associated with overall survival. A novel nine-gene signature predicting overall survival of pancreatic cancer was established via Lasso-Cox regression. MCOLN3 and SLC25A45 were downregulated and identified as protective genes whereas MET, KLK10, COL17A1, CEP55, ANKRD22, ITGB6, and ARNTL2 were upregulated and associated with poor survival. This nine-gene signature was an independent prognostic factor of pancreatic cancer. Patients in low-risk groups had significantly better prognoses than those in high-risk groups. Prognostic performance of the nine-gene signature was validated in the TCGA dataset and the external datasets GSE62452 and GSE57495. The AUC, C-index, and calibration curves confirmed that the nine-gene signature was comparable or superior to AJCC staging at predicting 1-, 2-, and 3-year overall survival. A nomogram integrated with the nine-gene signature and clinicopathological parameters was established and accurately predicted overall survival. GSEA disclosed that 23 oncological signatures were significantly enriched in the high-risk group defined by the nine-gene signature. This group was enriched with pancreatic cancer-related oncogenic pathways and mutations and was associated with invasion, metastasis, poor survival, and significantly lower levels of CD4^+^ T cell infiltration. As a supplement to AJCC staging, the nine-gene signature and the nomogram may be useful as progression indicators and predictors of overall survival.

Five of the genes in the nine-gene signature were previously reported to be associated with pancreatic cancer. MET is a receptor tyrosine kinase that transduces signals from the extracellular matrix to the cytoplasm by binding its ligand HGF. MET is dysregulated in pancreatic cancer and activated by genetic mutation and gene amplification, participating in pancreatic cancer cell interactions with the tumor microenvironment (29). It establishes the pre-metastatic microenvironment promoting the metastatic phenotype. MET expression is closely associated with clinical stage and activates the RAS-ERK and PI3K-Akt pathways by recruiting downstream effector molecules mediating tumorigenesis, progression, metastasis, and gemcitabine chemotherapy resistance. KLK10 is a serine protease and a member of the kallikrein family. Human tissue kallikreins regulate cancer cell growth, angiogenesis, invasion and metastasis, and either promote or suppress cancer (30). They are also used as cancer biomarkers. KLK10 is downregulated in breast, prostate, and other cancers functioning as a tumor suppressor. In contrast, KLK10 is upregulated in thyroid, gastric, and colorectal cancers and promotes tumors. In pancreatic cancer, KLK10 is highly expressed in pancreatic intraepithelial neoplasia and cancer tissues. KLK10 is upregulated in pancreatic cancer patients with lymph node involvement and remote metastasis (31). KLK10 and KLK6 are co-expressed in pancreatic cancer tissues, positively correlated with R1-resection status and poor prognosis and are independent risk factors (32). KLK10 knockdown attenuated pancreatic cancer cell migration, invasion, and metastasis *in vitro* and *in vivo*. KLK10 also mediates pancreatic cancer invasion and metastasis by activating the FAK-SRC-ERK signaling pathway. COL17A1 maintains hemidesmosome integrity and is the direct target of autoantibody in bullous dermatosis. In breast cancer, COL17A1 is hypermethylated and downregulated (33). Downregulation of COL17A1 is associated with poor prognosis in this case. COL17A1 may be a target of wild type p53 in breast tissue. It inhibits cell migration and invasion. In contrast, COL17A1 is hypomethylated and upregulated in cervical cancer, head, neck, and lung squamous cell carcinoma, and lung adenocarcinoma (34). COL17A1 upregulation was associated with poor prognosis in these cancers. COL17A1 enhances invasive squamous cell carcinoma migration and invasion via the FAK/PI3K signal pathway. Thus, COL17A1 may play a dual role in certain cancers. COL17A1 is upregulated in pancreatic cancer and positively associated with poor prognosis (35). However, the mechanisms by which it promotes pancreatic cancer have not been clarified. The methylation levels and specific mechanisms by which COL17A1 promotes pancreatic cancer under various p53 mutation states merit further investigation. CEP55 recruits PDCD6IP and TSG101, playing an important role in cytokinesis. CEP55 is upregulated in pancreatic cancer and is associated with poor survival (36). CEP55 upregulation induces invasion-related matrix metalloproteinase (MMP) and proliferation-related cyclin D1. CEP55 promotes pancreatic cancer proliferation, migration, and invasion *in vitro* and *in vivo* by activating the NF-κB signaling pathway and the PI3K/AKT signaling pathway. CEP55 was also reported to be upregulated in gastric, liver, lung, nasopharyngeal, and bladder cancers (37). Upregulation of CEP55 activates the PI3K/AKT signaling pathway in a concentration-dependent manner and promotes tumor proliferation, invasion, and metastasis. Its expression level is closely related to clinical stage and poor prognosis. Therefore, CEP55 is considered an ideal predictor of cancer prognosis. The biological function of ANKRD22 has not yet been fully elucidated. It was thought to be associated with the transition steps of somatic reprogramming, human ovulatory cascade, and T cell-mediated allograft rejection (38). Transcriptional profiling of peripheral blood in pancreatic cancer patients revealed that ANKRD22 mRNA was upregulated and could serve as a diagnostic biomarker in patients with AUC = 0.933 (39). In non-small cell lung cancer (NSCLC), ANKRD22 was upregulated in the tumor and correlated with relapse and overall survival. It promotes NSCLC proliferation by upregulating E2F1 transcription (40). The role of ANKRD22 in pancreatic cancer progression requires further study.

The roles of ITGB6 and ARNTL2 in pancreatic cancer have not been reported. ITGB6 forms a complex with ITGA5, which is the receptor for fibronectin and cytokinin. The ITGA5B6 complex recognizes the RGD sequence, mediating cell adhesion, and RGD-dependent TGFB1 release (41). ITGB6 was upregulated and promoted invasion and metastasis in multiple cancers. Internalization of the ITGA5B6 complex promotes cancer cell invasion. ITGB6 upregulation in breast cancer was associated with poor survival and metastasis. Anti-ITGA5B6 antibody alone or with trastuzumab halted tumor growth (42). ITGB6 was also considered as a novel serum marker and a highly efficient target for immunoliposome-mediated drug delivery in colon cancer (43). ARNTL2 is a transcriptional activator and a core component of the circadian clock. Interruption of the circadian rhythm induces cardiovascular disease, cancers, metabolic syndromes, and aging. Variations of the genes governing the circadian pathway may be associated with cancer predisposition. ARNTL2 participates in tumor progression. It is deregulated in B leukemia and repressed by RelB and RelA in EBV-transformed B cells (44). It is associated with colorectal and breast cancer invasiveness, metastasis, and aggressiveness (45). It induces a complex prometastatic secretome and enables self-sufficient lung adenocarcinoma metastasis (46). Invasion and metastasis are common and occur early in pancreatic cancer. The roles of ARTL22 in these processes deserve further investigation.

The roles of MCOLN3 and SLC25A45 in cancer development have not yet been elucidated. MCOLN3 is a non-selective ligand-gated cation channel that regulates membrane trafficking and mediates Ca^2+^ release from the endosome to the cytoplasm (47). Current research on MCOLN3 focuses on sensory modalities. MCOLN3 resides mainly in endosome membranes, regulates autophagy, and may participate in autophagosome formation (48). Autophagy promotes and suppresses cancer occurrence and progression. Therefore, MCOLN3 may mediate autophagy in these processes. SLC25A45 is a transport protein in the mitochondrial membrane, containing active thyroid-responsive elements (49). SLC25A45 mutations are associated with chronic kidney disease and preterm birth (50). The SNP of SLC25A45 was associated with the mucinous histological subtype of epithelial ovarian cancer (51). Although MCOLN3 and SLC25A45 have not been studied intensively in cancer, data from the TCGA database revealed that MCOLN3 was downregulated in adrenocortical (ACC), breast invasive (BRCA), uterine corpus endometrial (UCEC), kidney renal clear cell (KIRC), and kidney renal papillary cell (KIRP) carcinomas, colon (COAD), lung (LUAD), lung squamous cell (LUSC), rectal (READ), and stomach (STAD) adenocarcinomas, pheochromocytoma, and paraganglioma (PCPG), thymoma (THYM), and uterine carcinosarcoma (UCS) (|LOG2FC| > 1 and *P* < 0.01) and was associated with relatively better survival in KIRC (HR <1 and *P* < 0.05). SLC25A45 was downregulated in lower grade glioma (LGG), LUSC, and thyroid carcinoma (THCA). The roles of MCOLN3 and SLC25A45 in cancer are worthy of further investigation.

Immunotherapy is currently a routine cancer treatment option. CD8^+^ cytotoxic T lymphocytes recognize MHC I-presenting antigens and are preferred for targeting tumor cells. On the other hand, CD4^+^ T lymphocytes play complex and important roles in tumor immunity. It is generally considered that CD4^+^ T cells compromise the majority of T cells in pancreatic cancer and are positively associated with metastasis and negatively associated with survival (52). The pioneer study of Zhang et al. further revealed that Kras-driven oncogenesis of pancreatic cancer established an immunosuppressive microenvironment via recruitment and activity of CD4^+^ T lymphocytes (53). Elimination of CD4^+^ T lymphocytes restored the antitumor function of CD8^+^ T lymphocytes and blocked carcinogenesis. The specific subsets of CD4^+^ T lymphocytes that play major immunosuppressive role remain to be elucidated. On the other hand, certain subsets of CD4^+^ T lymphocytes may also be needed for antitumor immunity. CD4^+^ helper T cells may promote and maintain cytotoxic T lymphocyte (CTL) memory, amplify T- and B cells, and help CTL overcome negative regulation (54). CD4^+^ T lymphocytes may eliminate tumor cells by cytolysis or by regulating the tumor microenvironment (55). In the current study, results calculated by algorithms indicated that CD4^+^ T lymphocyte infiltration was significantly downregulated in high-risk tumor tissues and was associated with poor prognosis. These results require verification by further experimental studies. Considering the differential infiltration level of immune cells between high-risk and low-risk groups of pancreatic cancer, high-risk patients may benefit from more accurate immunotherapy strategies. More detailed studies are also required to elucidate the specific role of each CD4^+^ T lymphocyte subset in order to enhance the antitumor efficacy of CD8^+^ cytotoxic T lymphocyte.

Our predictive model is based on the expression levels of genes in a selected panel. This approach is more economical and clinically practical than whole-genome sequencing. Our nomogram incorporating nine-gene signature and clinicopathological parameters may enable clinicians to determine individual patient's prognosis. Its graphical scoring system is easy to understand facilitating the customized treatment and making of medical decisions. To the best of our knowledge, the nine-gene prognostic signature described herein and the nomogram based on it have not been reported previously. Three previously defined prognostic signatures with published algorithms were used as controls in the current study. Yan et al. identified a four-gene signature (LYRM1, KNTC1, IGF2BP2, and CDC6) significantly associated with progression and prognosis of pancreatic cancer (8). Chen et al. proposed a 3-gene signature (SULT1E1, IGF2BP3, and MAP4K4) based on DNA methylation data that predicts poorer overall survival of pancreatic cancer (23). Liao et al. reported a nine-gene prognostic model (ARHGAP30, HCLS1, CD96, FAM78A, ARHGAP15, SLA2, CD247, GVINP1, and IL16) using weighted gene co-expression network analysis that may predict overall survival of pancreatic cancer patients after pancreaticoduodenectomy (24).These articles explored prognosis-related genes from different perspectives to establish prognostic signatures. No overlap was identified between the nine-gene prognostic signature we developed and the one previously defined. Our prognostic signature was identified to be superior or comparable to the previous defined signatures. Our study provides new insight into the molecular mechanism of pancreatic cancer and prediction of prognosis. Moreover, the DEGs obtained in this study were derived from the integrated analysis of multiple datasets, which is highly reliable. Four of the genes in the nine-gene signature had not yet been reported to be associated with pancreatic cancer prior to this study. These DEGs may be potential molecular targets to fight pancreatic cancer.

However, the present study had certain limitations. First, the main sources of our clinical information were datasets from the TCGA and GEO databases. Most of the patients therein are Whites, Africans, or Latinos. Caution must be taken when extrapolating our findings to patients from other ethnicities. The current study is driven by statistics of available retrospective data and the optimal cutoff is required to be determined before clinical application. Second, the establishment and verification of the nomogram were based on the TCGA database. Therefore, it will be necessary to verify using external datasets with complete clinical information and gene expression information in the future. Moreover, the protein expression levels of the prognosis related DEGs and their molecular mechanisms in pathogenesis and progression of pancreatic cancer depend on further experimental studies to elucidate.

## Conclusion

Our study identified a nine-gene signature and a prognostic nomogram incorporating the gene signature and clinical prognostic factors to predict overall survival of pancreatic cancer. The nine-gene signature was closely associated with the progression, aggressiveness, and prognosis of pancreatic cancer and its constituents are potential therapeutic targets. The prognostic nomogram reliably predicted overall survival in pancreatic cancer and may facilitate individualized treatment and making of medical decisions.

## Data Availability Statement

The datasets analyzed for this study can be found in the Gene Expression Omnibus (https://www.ncbi.nlm.nih.gov/geo/) and TCGA (https://portal.gdc.cancer.gov/).

## Author Contributions

ZL and YZ: conception and design, and study supervision. MW and XL: development of methodology, analysis and interpretation of data, and writing of the manuscript. ZL, TZ, and YZ: review of the manuscript.

### Conflict of Interest

The authors declare that the research was conducted in the absence of any commercial or financial relationships that could be construed as a potential conflict of interest.

## Supplementary Material

Supplementary Table 1 234 DEGs identified by integrated analysis using Robust rank aggregation (RRA) method with *p* < 0.05.

Supplementary Table 2 Functional enrichment analysis of the DEGs.

Supplementary Table 3 DEGs associated with overall survival using univariate Cox regression model with *p* < 0.01.

Supplementary Table 4 Mean cross-validation error(CVM)and Standard deviation of CVM (CVSD) for each lambda in the LASSO regression.

Supplementary Table 5 The reasons for each case excluded from univariate- and multivariate Cox regression analyses.

Supplementary Table 6 Gene set enrichment analyses between high and low risk group in 165 TCGA PAAD Samples.

Supplementary Figure 1 Differential expression of mRNA between tumor and normal tissue in seven GEO datasets.

Supplementary Figure 2 The expression of DEGs identified after integrated analysis in GSE71729, GSE62165, GSE62452, GSE28735, GSE15471, and GSE32676.

Supplementary Figure 3 Functional enrichment analyses of the DEGs and the identification of hub genes.

Supplementary Figure 4 Lasso analysis of the prognostic DEGs in pancreatic cancer.

Supplementary Figure 5 ROC curves for overall survival predictions of the nine gene signature in compare with 3 previously defined gene signatures.

Supplementary Figure 6 Subgroup analyses of the nine gene signature.

Supplementary Figure 7 Analyses of response to treatment for patients in high risk and low risk group.

Supplementary Figure 8 External validation of the nine gene signature in GSE57495 dataset.

Supplementary Figure 9 Distribution of the risk score between metastases and the primary tumors and in different AJCC stages.

## References

[1] Ilic M Ilic I. Epidemiology of pancreatic cancer. World J Gastroenterol. (2016) 22:9694–705. 10.3748/wjg.v22.i44.9694 27956793 PMC5124974

[2] Rahib L Smith BD Aizenberg R Rosenzweig AB Fleshman JM Matrisian LM. Projecting cancer incidence and deaths to 2030: the unexpected burden of thyroid, liver, and pancreas cancers in the United States. Cancer Res. (2014) 74:2913–21. 10.1158/0008-5472.CAN-14-0155 24840647

[3] Kamisawa T Wood LD Itoi T Takaori K. Pancreatic cancer. Lancet. (2016) 388:73–85. 10.1016/S0140-6736(16)00141-0 26830752

[4] Singhi AD Koay EJ Chari ST Maitra A. Early detection of pancreatic cancer: opportunities and challenges. Gastroenterology. (2019) 156:2024–40. 10.1053/j.gastro.2019.01.259 30721664 PMC6486851

[5] Kamarajah SK Burns WR Frankel TL Cho CS Nathan H. Validation of the American Joint Commission on Cancer (AJCC) 8th edition staging system for patients with pancreatic adenocarcinoma: a surveillance, epidemiology and end results (SEER) analysis. Ann Surg Oncol. (2017) 24:2023–30. 10.1245/s10434-017-5810-x 28213792

[6] Birnbaum DJ Finetti P Lopresti A Gilabert M Poizat F Raoul JL. A 25-gene classifier predicts overall survival in resectable pancreatic cancer. BMC Med. (2017) 15:170. 10.1186/s12916-017-0936-z 28927421 PMC5606023

[7] Raman P Maddipati R Lim KH Tozeren A. Pancreatic cancer survival analysis defines a signature that predicts outcome. PLoS ONE. (2018) 13:e0201751. 10.1371/journal.pone.0201751 30092011 PMC6084949

[8] Yan X Wan H Hao X Lan T Li W Xu L. Importance of gene expression signatures in pancreatic cancer prognosis and the establishment of a prediction model. Cancer Manag Res. (2019) 11:273–83. 10.2147/CMAR.S185205 30643453 PMC6312063

[9] Moffitt RA Marayati R Flate EL Volmar KE Loeza SG Hoadley KA. Virtual microdissection identifies distinct tumor- and stroma-specific subtypes of pancreatic ductal adenocarcinoma. Nat Genet. (2015) 47:1168–78. 10.1038/ng.3398 26343385 PMC4912058

[10] Janky R Binda MM Allemeersch J Van den Broeck A Govaere O Swinnen JV. Prognostic relevance of molecular subtypes and master regulators in pancreatic ductal adenocarcinoma. BMC Cancer. (2016) 16:632. 10.1186/s12885-016-2540-6 27520560 PMC4983037

[11] Yang S He P Wang J Schetter A Tang W Funamizu N. A novel MIF signaling pathway drives the malignant character of pancreatic cancer by targeting NR3C2. Cancer Res. (2016) 76:3838–50. 10.1007/978-3-319-42740-9 27197190 PMC4930741

[12] Zhang G He P Tan H Budhu A Gaedcke J Ghadimi BM. Integration of metabolomics and transcriptomics revealed a fatty acid network exerting growth inhibitory effects in human pancreatic cancer. Clin Cancer Res. (2013) 19:4983–93. 10.1158/1078-0432.CCR-13-0209 23918603 PMC3778077

[13] Badea L Herlea V Dima SO Dumitrascu T Popescu I. Combined gene expression analysis of whole-tissue and microdissected pancreatic ductal adenocarcinoma identifies genes specifically overexpressed in tumor epithelia. Hepatogastroenterology. (2008) 55:2016–27. 19260470

[14] Pei H Li L Fridley BL Jenkins GD Kalari KR Lingle W. FKBP51 affects cancer cell response to chemotherapy by negatively regulating Akt. Cancer Cell. (2009) 16:259–66. 10.1016/j.ccr.2009.07.016 19732725 PMC2755578

[15] Donahue TR Tran LM Hill R Li Y Kovochich A Calvopina JH. Integrative survival-based molecular profiling of human pancreatic cancer. Clin Cancer Res. (2012) 18:1352–63. 10.1158/1078-0432.CCR-11-1539 22261810 PMC3816537

[16] Chen DT Davis-Yadley AH Huang PY Husain K Centeno BA Permuth-Wey J. Prognostic fifteen-gene signature for early stage pancreatic ductal adenocarcinoma. PLoS ONE. (2015) 10:e0133562. 10.1371/journal.pone.0133562 26247463 PMC4527782

[17] Tang Z Li C Kang B Gao G Li C Zhang Z. GEPIA: a web server for cancer and normal gene expression profiling and interactive analyses. Nucleic Acids Res. (2017) 45:W98–102. 10.1093/nar/gkx247 28407145 PMC5570223

[18] Uhlen M Fagerberg L Hallstrom BM Lindskog C Oksvold P Mardinoglu A. Proteomics. Tissue-based map of the human proteome. Science. (2015) 347:1260419. 10.1126/science.1260419 25613900

[19] Gao J Aksoy BA Dogrusoz U Dresdner G Gross B Sumer SO. Integrative analysis of complex cancer genomics and clinical profiles using the cBioPortal. Sci Signal. (2013) 6:pl1. 10.1126/scisignal.2004088 23550210 PMC4160307

[20] Huang da W Sherman BT Lempicki RA. Systematic and integrative analysis of large gene lists using DAVID bioinformatics resources. Nat Protoc. (2009) 4:44–57. 10.1038/nprot.2008.211 19131956

[21] Szklarczyk D Franceschini A Wyder S Forslund K Heller D Huerta-Cepas J. STRING v10: protein-protein interaction networks, integrated over the tree of life. Nucleic acids research (2015) 43(Database issue):D447–52. Epub 2014/10/30. 10.1093/nar/gku1003 25352553 PMC4383874

[22] Camp RL Dolled-Filhart M Rimm DL. X-tile: a new bio-informatics tool for biomarker assessment and outcome-based cut-point optimization. Clin Cancer Res. (2004) 10:7252–9. 10.1158/1078-0432.CCR-04-0713 15534099

[23] Chen H Kong Y Yao Q Zhang X Fu Y Li J. Three hypomethylated genes were associated with poor overall survival in pancreatic cancer patients. Aging. (2019) 11:885–97. 10.18632/aging.101785 30710069 PMC6382432

[24] Liao X Huang K Huang R Liu X Han C Yu L. Genome-scale analysis to identify prognostic markers in patients with early-stage pancreatic ductal adenocarcinoma after pancreaticoduodenectomy. OncoTargets Ther. (2017) 10:4493–506. 10.2147/OTT.S142557 28979141 PMC5602474

[25] Subramanian A Tamayo P Mootha VK Mukherjee S Ebert BL Gillette MA. Gene set enrichment analysis: a knowledge-based approach for interpreting genome-wide expression profiles. Proc Natl Acad Sci USA. (2005) 102:15545–50. 10.1073/pnas.0506580102 16199517 PMC1239896

[26] Yoshihara K Shahmoradgoli M Martinez E Vegesna R Kim H Torres-Garcia W. Inferring tumour purity and stromal and immune cell admixture from expression data. Nat Commun. (2013) 4:2612. 10.1038/ncomms3612 24113773 PMC3826632

[27] Li T Fan J Wang B Traugh N Chen Q Liu JS. TIMER: A web server for comprehensive analysis of tumor-infiltrating immune cells. Cancer Res. (2017) 77:e108–10. 10.1158/0008-5472.CAN-17-0307 29092952 PMC6042652

[28] Balachandran VP Gonen M Smith JJ DeMatteo RP. Nomograms in oncology: more than meets the eye. Lancet Oncol. (2015) 16:e173–80. 10.1016/S1470-2045(14)71116-7 25846097 PMC4465353

[29] Pothula SP Xu Z Goldstein D Merrett N Pirola RC Wilson JS. Targeting the HGF/c-MET pathway: stromal remodelling in pancreatic cancer. Oncotarget (2017) 8:76722–39. Epub 2017/11/05. 10.18632/oncotarget.20822 29100344 PMC5652738

[30] Filippou PS Karagiannis GS Musrap N Diamandis EP. Kallikrein-related peptidases (KLKs) and the hallmarks of cancer. Crit Rev Clin Lab Sci. (2016) 53:277–91. 10.3109/10408363.2016.1154643 26886390

[31] Cao XY Zhang XX Yang MW Hu LP Jiang SH Tian GA. Aberrant upregulation of KLK10 promotes metastasis via enhancement of EMT and FAK/SRC/ERK axis in PDAC. Biochem Biophys Res Commun. (2018) 499:584–93. 10.1016/j.bbrc.2018.03.194 29621546

[32] Sproule TJ Bubier JA Grandi FC Sun VZ Philip VM McPhee CG. Molecular identification of collagen 17a1 as a major genetic modifier of laminin gamma 2 mutation-induced junctional epidermolysis bullosa in mice. PLoS Genet. (2014) 10:e1004068. 10.1371/journal.pgen.1004068 24550734 PMC3923665

[33] Yodsurang V Tanikawa C Miyamoto T Lo PHY Hirata M Matsuda K. Identification of a novel p53 target, COL17A1, that inhibits breast cancer cell migration and invasion. Oncotarget. (2017) 8:55790–803. 10.18632/oncotarget.18433 28915553 PMC5593524

[34] Thangavelu PU Krenacs T Dray E Duijf PH. In epithelial cancers, aberrant COL17A1 promoter methylation predicts its misexpression and increased invasion. Clin Epigenet. (2016) 8:120. 10.1186/s13148-016-0290-6 27891193 PMC5116176

[35] Wu J Li Z Zeng K Wu K Xu D Zhou J. Key genes associated with pancreatic cancer and their association with outcomes: a bioinformatics analysis. Mol Med Rep. (2019) 20:1343–52. 10.3892/mmr.2019.10321 31173193

[36] Peng T Zhou W Guo F Wu HS Wang CY Wang L. Centrosomal protein 55 activates NF-kappaB signalling and promotes pancreatic cancer cells aggressiveness. Sci Rep. (2017) 7:5925. 10.1038/s41598-017-06132-z 28724890 PMC5517556

[37] Jeffery J Sinha D Srihari S Kalimutho M Khanna KK. Beyond cytokinesis: the emerging roles of CEP55 in tumorigenesis. Oncogene. (2016) 35:683–90. 10.1038/onc.2015.128 25915844

[38] Halloran PF Venner JM Madill-Thomsen KS Einecke G Parkes MD Hidalgo LG. Review: the transcripts associated with organ allograft rejection. Am J Transplant. (2018) 18:785–95. 10.1111/ajt.14600 29178397

[39] Caba O Prados J Ortiz R Jimenez-Luna C Melguizo C Alvarez PJ. Transcriptional profiling of peripheral blood in pancreatic adenocarcinoma patients identifies diagnostic biomarkers. Dig Dis Sci. (2014) 59:2714–20. 10.1007/s10620-014-3291-3 25069573

[40] Yin J Fu W Dai L Jiang Z Liao H Chen W. ANKRD22 promotes progression of non-small cell lung cancer through transcriptional up-regulation of E2F1. Sci Rep. (2017) 7:4430. 10.1038/s41598-017-04818-y 28667340 PMC5493668

[41] Dong X Zhao B Iacob RE Zhu J Koksal AC Lu C. Force interacts with macromolecular structure in activation of TGF-beta. Nature. (2017) 542:55–9. 10.1038/nature21035 28117447 PMC5586147

[42] Moore KM Thomas GJ Duffy SW Warwick J Gabe R Chou P. Therapeutic targeting of integrin alphavbeta6 in breast cancer. J Natl Cancer Inst. (2014) 106:dju169. 10.1093/jnci/dju169 24974129 PMC4151855

[43] Bengs S Becker E Busenhart P Spalinger MR Raselli T Kasper S. beta6 -integrin serves as a novel serum tumor marker for colorectal carcinoma. Int J Cancer. (2019) 145:678–85. 10.1002/ijc.32137 30653264

[44] Chanut A Duguet F Marfak A David A Petit B Parrens M. RelA and RelB cross-talk and function in Epstein-Barr virus transformed B cells. Leukemia. (2014) 28:871–9. 10.1038/leu.2013.274 24056880

[45] Ha NH Long J Cai Q Shu XO Hunter KW. The circadian rhythm gene Arntl2 is a metastasis susceptibility gene for estrogen receptor-negative breast cancer. PLoS Genet. (2016) 12:e1006267. 10.1371/journal.pgen.1006267 27656887 PMC5033489

[46] Brady JJ Chuang CH Greenside PG Rogers ZN Murray CW Caswell DR. An Arntl2-driven secretome enables lung adenocarcinoma metastatic self-sufficiency. Cancer Cell. (2016) 29:697–710. 10.1016/j.ccell.2016.03.003 27150038 PMC4864124

[47] Kim HJ Soyombo AA Tjon-Kon-Sang S So I Muallem S. The Ca^(2+)^ channel TRPML3 regulates membrane trafficking and autophagy. Traffic. (2009) 10:1157–67. 10.1111/j.1600-0854.2009.00924.x 19522758 PMC2993507

[48] Kim SW Kim DH Park KS Kim MK Park YM Muallem S Palmitoylation controls trafficking of the intracellular Ca^(2+)^ channel MCOLN3/TRPML3 to regulate autophagy. Autophagy. (2019) 15:327–40. 10.1080/15548627.2018.1518671 30215288 PMC6333453

[49] Paquette MA Dong H Gagne R Williams A Malowany M Wade MG. Thyroid hormone-regulated gene expression in juvenile mouse liver: identification of thyroid response elements using microarray profiling and in silico analyses. BMC Genom. (2011) 12:634. 10.1186/1471-2164-12-634 22206413 PMC3340398

[50] Uzun A Sahin Y Schuster JS Zheng X Ryckman K Feingold E. Structural and genomic variation in preterm birth. Pediatr Res. (2016) 80:829–36. 10.1038/pr.2016.152 27466897 PMC5112111

[51] Chornokur G Lin HY Tyrer JP Lawrenson K Dennis J Amankwah EK. Common genetic variation in cellular transport genes and epithelial ovarian cancer (EOC) risk. PLoS ONE. (2015) 10:e0128106. 10.1371/journal.pone.0128106 26091520 PMC4474865

[52] Hiraoka N Onozato K Kosuge T Hirohashi S. Prevalence of FOXP3+ regulatory T cells increases during the progression of pancreatic ductal adenocarcinoma and its premalignant lesions. Clin Cancer Res. (2006) 12:5423–34. 10.1158/1078-0432.CCR-06-0369 17000676

[53] Zhang Y Yan W Mathew E Bednar F Wan S Collins MA. CD4^+^ T lymphocyte ablation prevents pancreatic carcinogenesis in mice. Cancer Immunol Res. (2014) 2:423–35. 10.1158/2326-6066.CIR-14-0016-T 24795355 PMC4160804

[54] Borst J Ahrends T Babala N Melief CJM Kastenmuller W. CD4^(+)^ T cell help in cancer immunology and immunotherapy. Nat Rev Immunol. (2018) 18:635–47. 10.1038/s41577-018-0044-0 30057419

[55] Kennedy R Celis E. Multiple roles for CD4^+^ T cells in anti-tumor immune responses. Immunol Rev. (2008) 222:129–44. 10.1111/j.1600-065X.2008.00616.x 18363998

