# Supplementary figures and images for: Identification of a Nine-Gene Signature and Establishment of a Prognostic Nomogram Predicting Overall Survival of Pancreatic Cancer
Source: Front Oncol. 2019 Sep 27;9:996. doi: 10.3389/fonc.2019.00996 (PMC6776930; doi:10.3389/fonc.2019.00996)

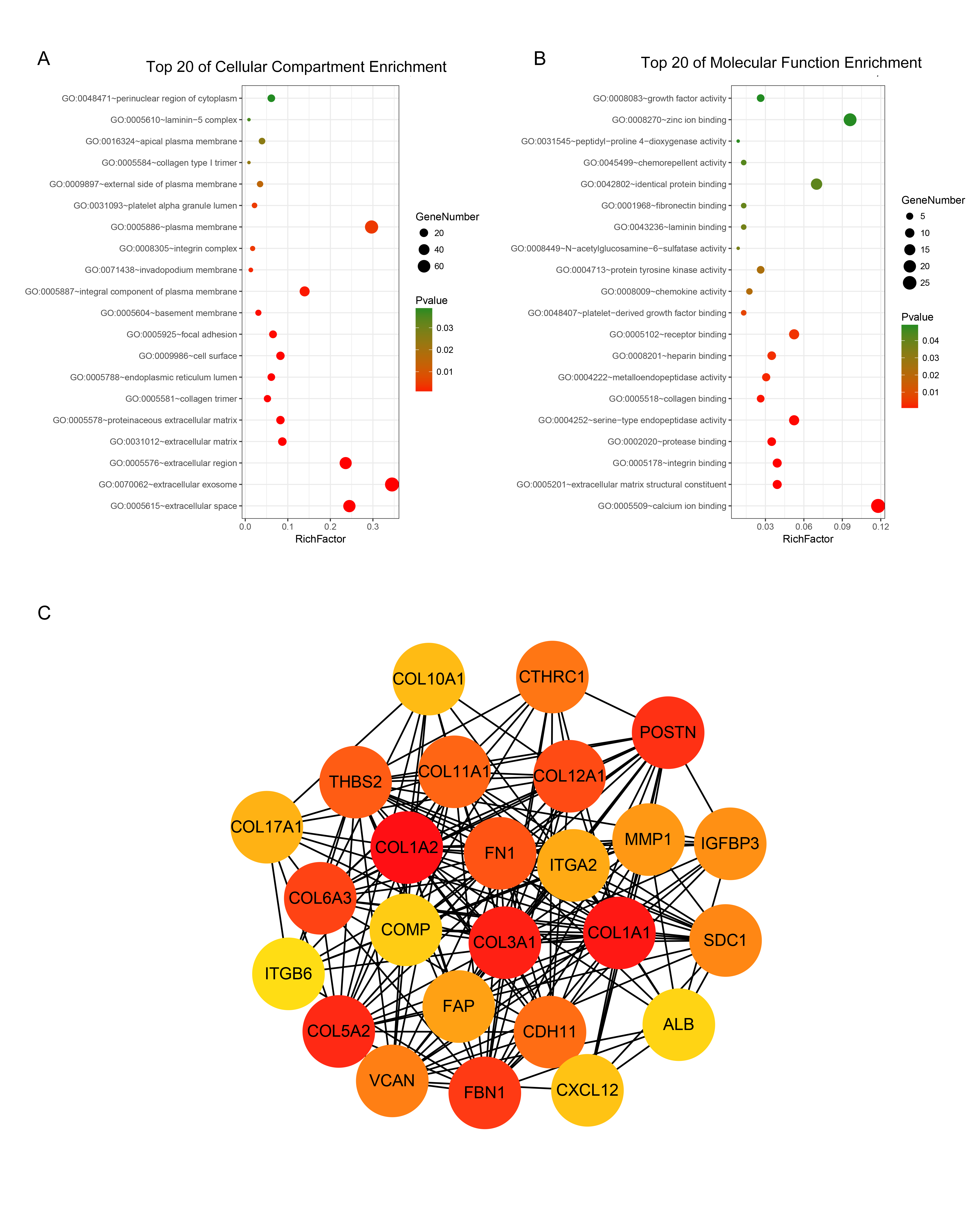

Supplement: Supplementary Figure 3 — Functional enrichment analyses of the DEGs and the identification of hub genes. [file Image_3.TIF]

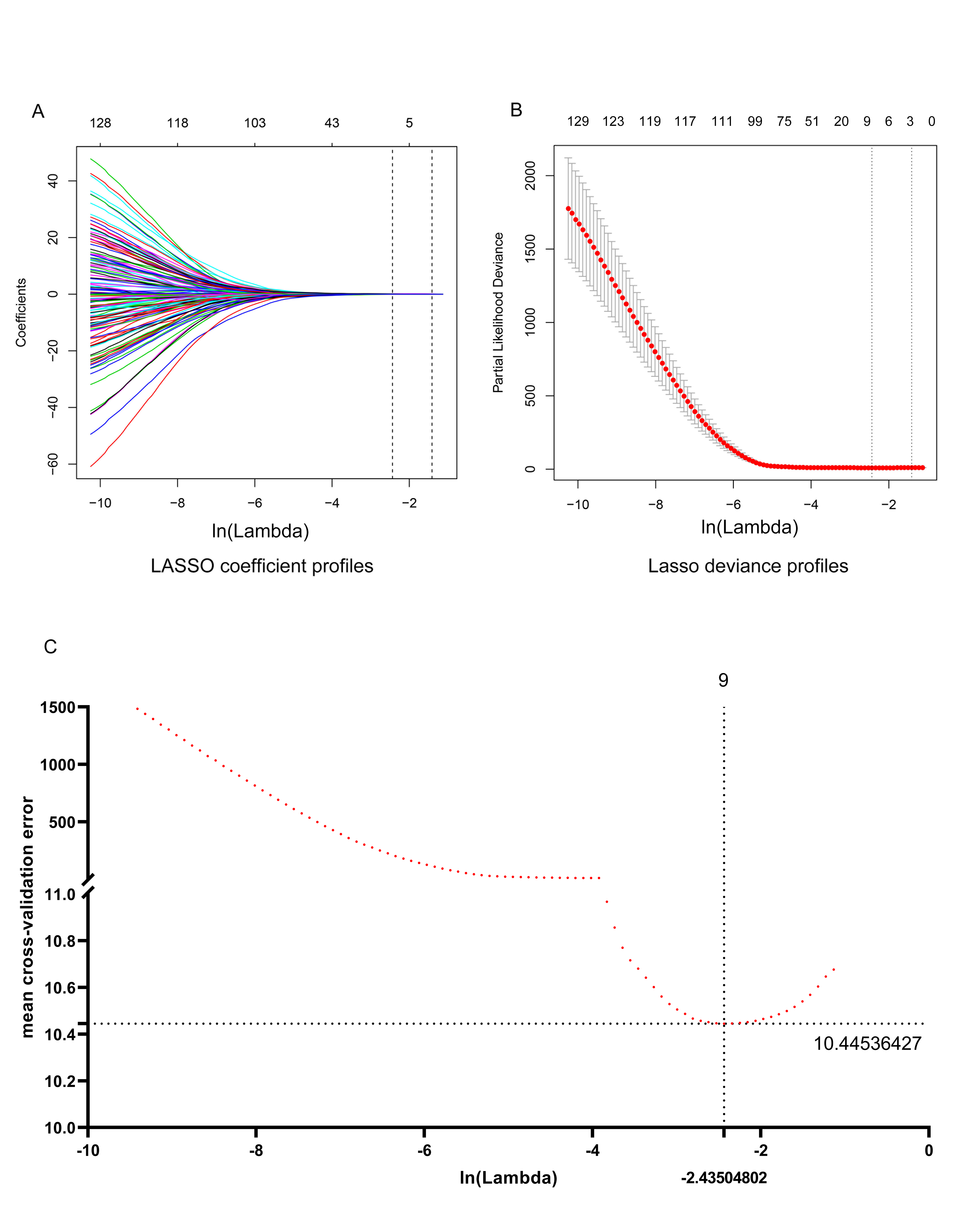

Supplement: Supplementary Figure 4 — Lasso analysis of the prognostic DEGs in pancreatic cancer. [file Image_4.TIF]

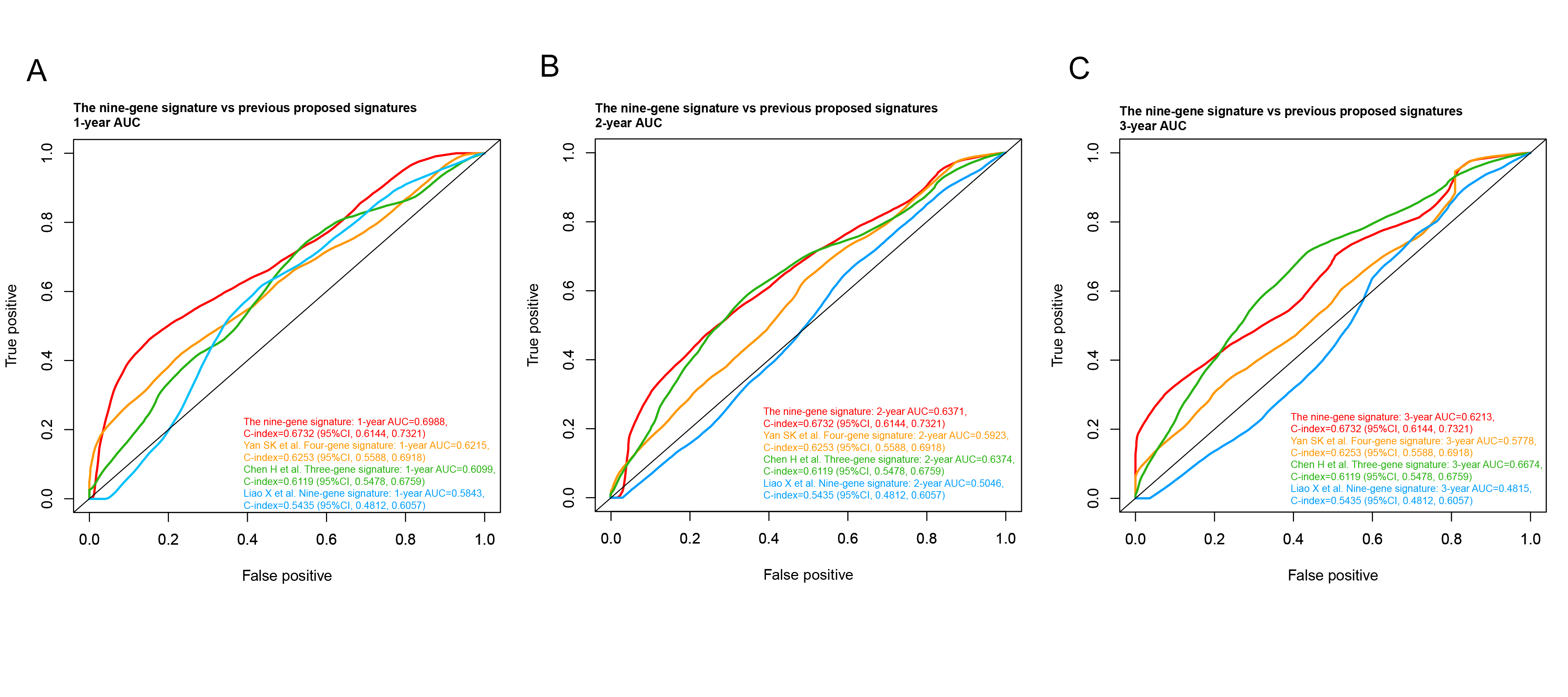

Supplement: Supplementary Figure 5 — ROC curves for overall survival predictions of the nine gene signature in compare with 3 previously defined gene signatures. [file Image_5.TIF]

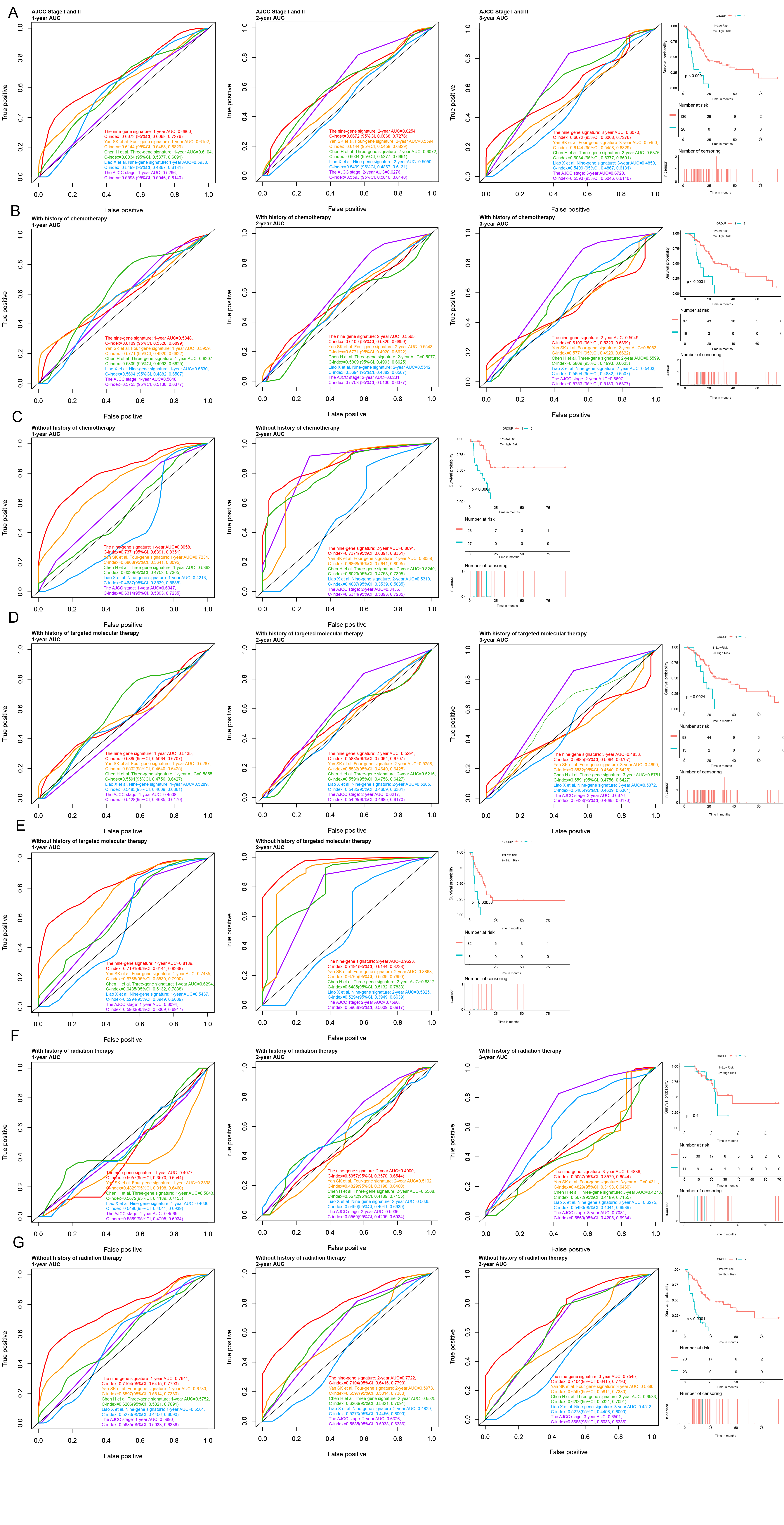

Supplement: Supplementary Figure 6 — Subgroup analyses of the nine gene signature. [file Image_6.TIF]

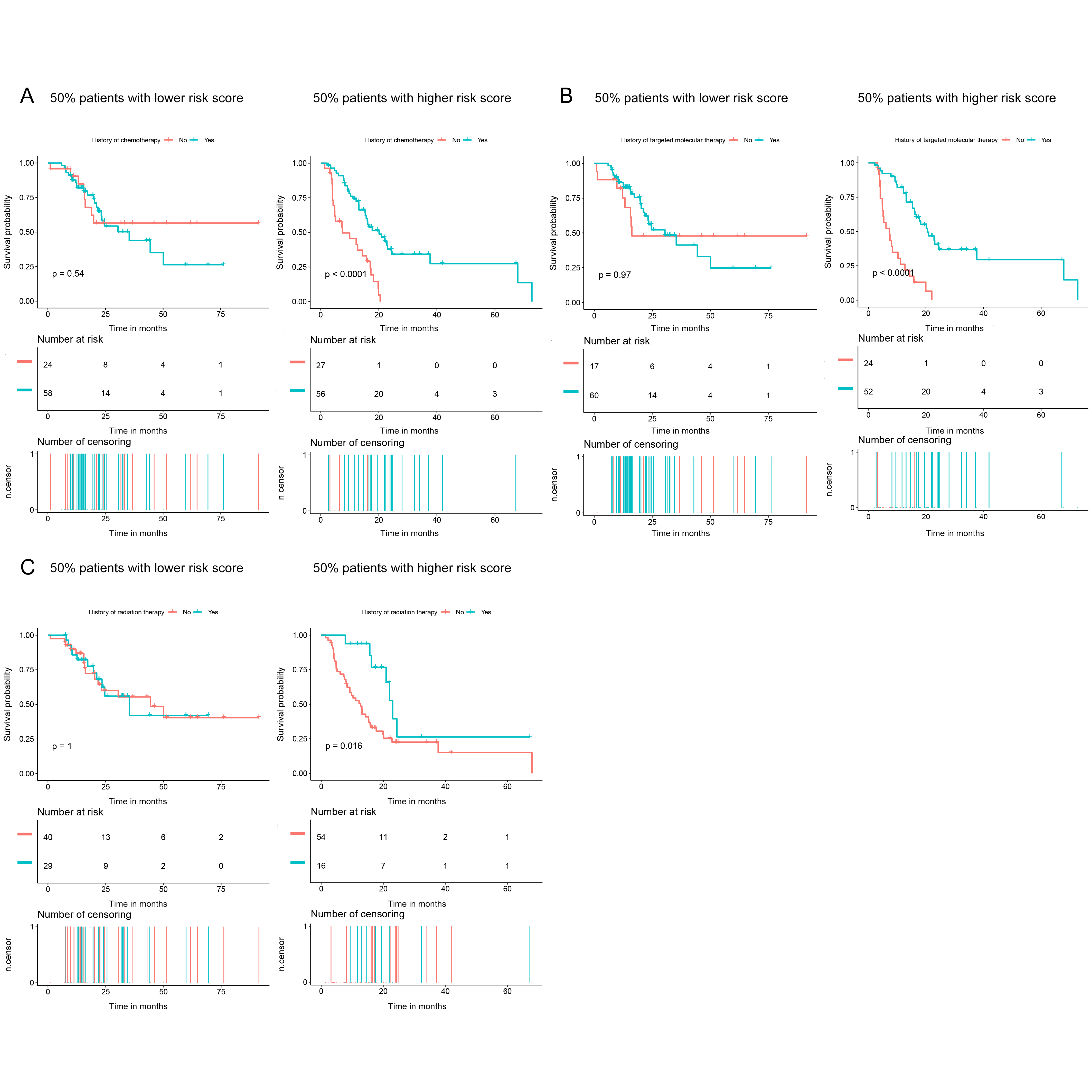

Supplement: Supplementary Figure 7 — Analyses of response to treatment for patients in high risk and low risk group. [file Image_7.TIF]

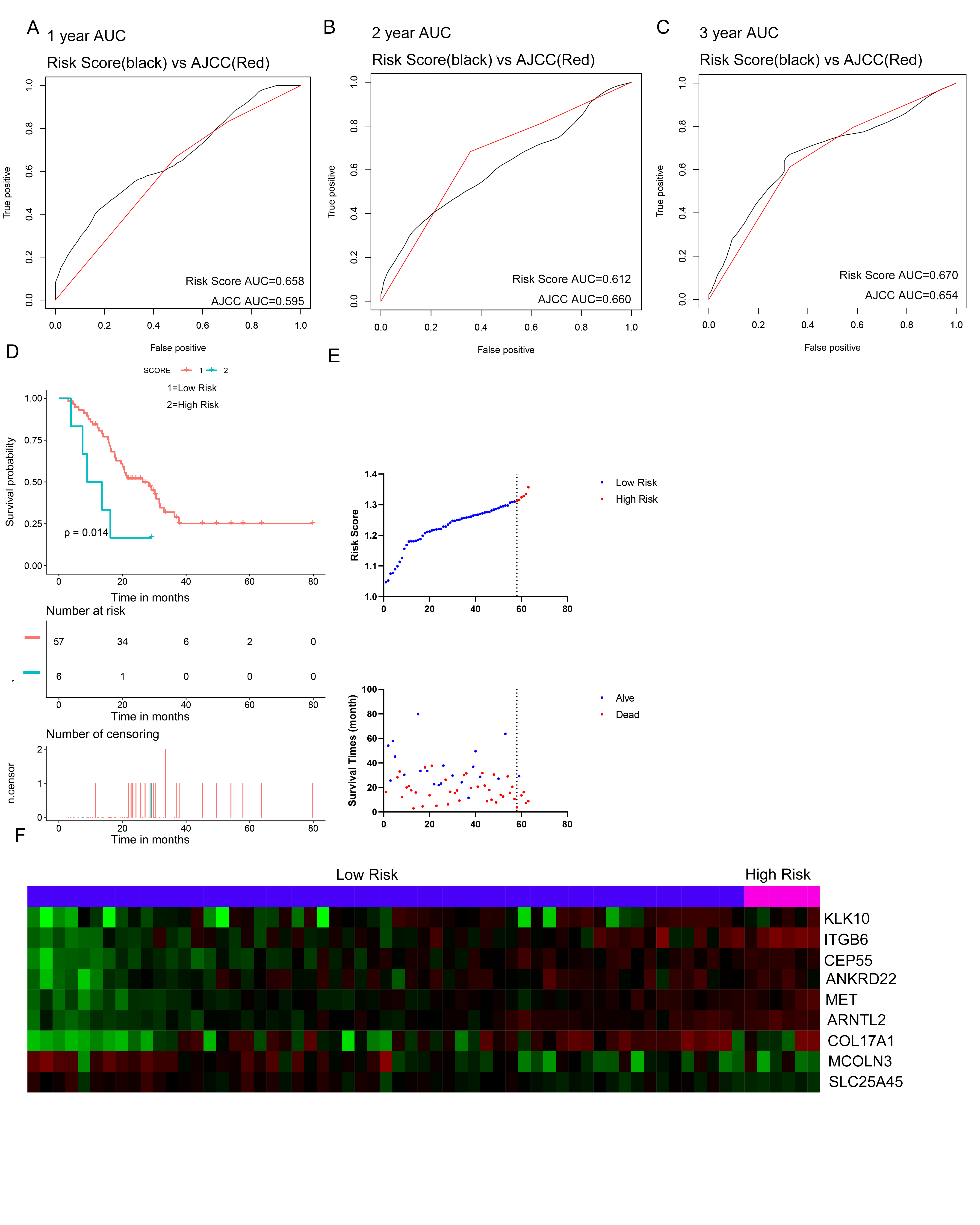

Supplement: Supplementary Figure 8 — External validation of the nine gene signature in GSE57495 dataset. [file Image_8.TIF]

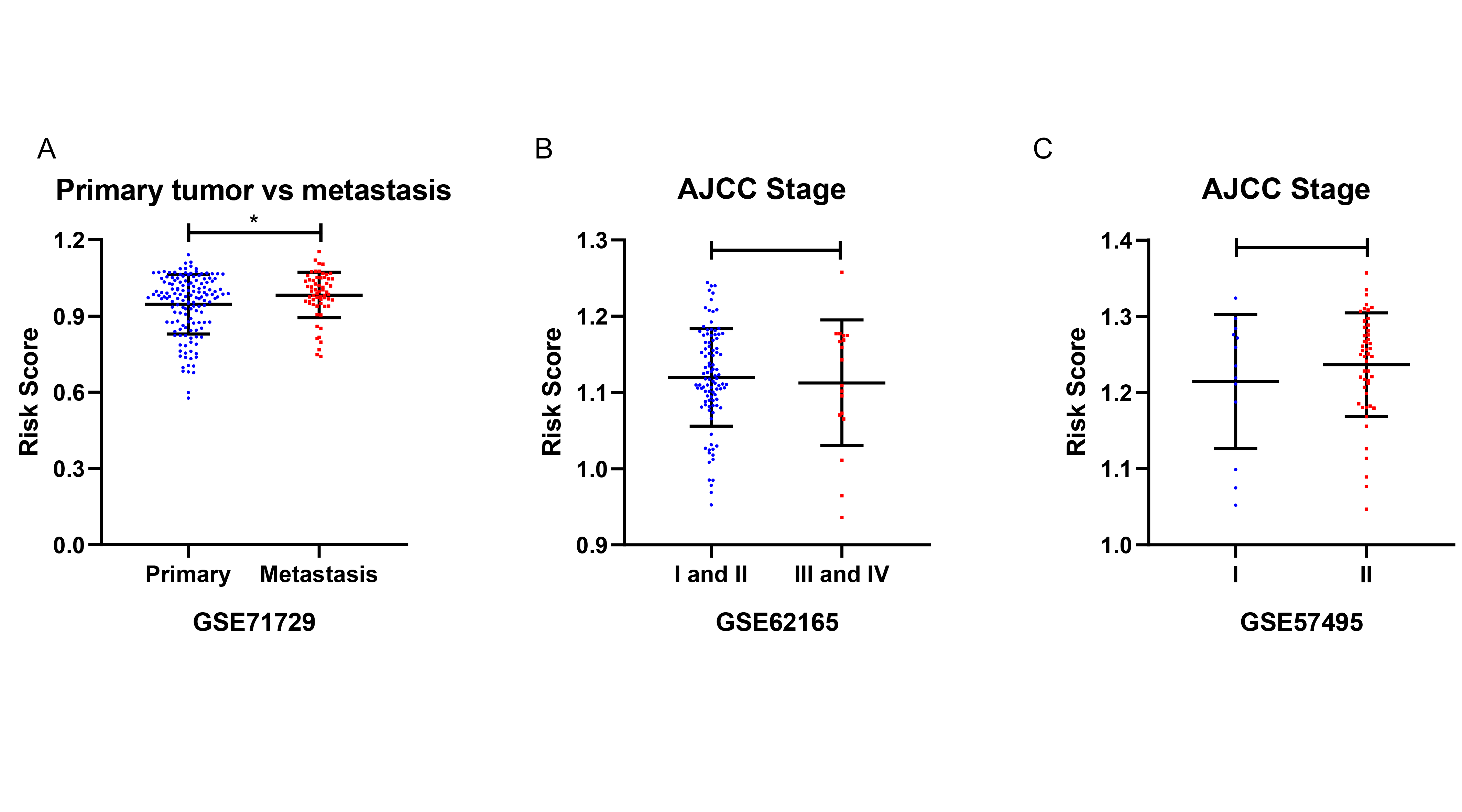

Supplement: Supplementary Figure 9 — Distribution of the risk score between metastases and the primary tumors and in different AJCC stages. [file Image_9.TIF]
